# Kojic Acid Derivative as an Antimitotic Agent That Selectively Kills Tumour Cells

**DOI:** 10.3390/ph18010011

**Published:** 2024-12-25

**Authors:** Giuseppina Pichiri, Marco Piludu, Terenzio Congiu, Nicole Grandi, Pierpaolo Coni, Monica Piras, Mariusz Jaremko, Joanna Izabela Lachowicz

**Affiliations:** 1 Department of Medical Sciences and Public Health, University of Cagliari, Cittadella Universitaria, 09042 Monserrato, Italy; pichiri@unica.it (G.P.); terenzio.congiu@unica.it (T.C.); coni@unica.it (P.C.); monica.piras@unica.it (M.P.); 2 Department of Biomedical Sciences, University of Cagliari, Cittadella Universitaria, 09042 Monserrato, Italy; mpiludu@unica.it; 3 Department of Life and Environmental Sciences, University of Cagliari, Cittadella Universitaria, 09042 Monserrato, Italy; nicole.grandi@unica.it; 4 Smart-Health Initiative (SHI) and Red Sea Research Center (RSRC), Division of Biological and Environmental Sciences and Engineering (BESE), King Abdullah University of Science and Technology (KAUST), Thuwal 23955-6900, Saudi Arabia; mariusz.jaremko@kaust.edu.sa; 5 Department of Population Health, Division of Environmental Health, Occupational Medicine and Epidemiology, Wroclaw Medical University, Mikulicza-Radeckiego 7, 50-368 Wroclaw, Poland

**Keywords:** mitosis, antimitotic agents, microscopy, next generation sequencing (NGS), cytoflow, apoptosis, cell cultures, selectivity

## Abstract

**Background/Objectives:** The primary method used to pharmacologically arrest cancer development and its metastasis is to disrupt the cell division process. There are a few approaches that may be used to meet this objective, mainly through inhibiting DNA replication or mitosis. Despite intensive studies on new chemotherapeutics, the biggest problem remains the side effects associated with the inhibition of cell division in non-tumoural host cells. **Methods:** The efficacy and selectivity of the kojic acid derivative (L1) was studied in vitro with the use of tumoural (Caco2, SW480, HT29, T98G) and non- tumoural (HEK293T, RAW) cell lines. Light and electron microscopy observations were supported by the next generation sequencing (NGS), cytoflow, and spectroscopy analysis of mRNA and biomolecules, respectively. **Results:** The light and electron microscopy observations showed that L1 treatment leads to significant morphological changes in Caco2 cells, which are characteristic of mitosis arrest. Moreover, the fluorescent tubulin staining revealed the formation of tubulin ring structure associated with the apoptotic stage. Mitotic exit into apoptosis was further conformed by the cytoflow of early/late apoptosis stages and caspase-3 analysis. NGS investigation showed differentiated expressions of genes involved in mitosis and apoptosis processes. The observed IC50 in tumoural cell lines were as follows: Caco2 (IC50 = 68.2 mM), SW480 (IC50 = 15.5 mM), and HT29 (IC50 = 4.7 mM). **Conclusions:** The findings presented here suggest that L1 could be a valid candidate for oral prevention and/or chemotherapy in colorectal cancer. Considering high selectivity of L1 versus tumoural cell lines, more in-depth mechanistic studies could reveal unknown stages in carcinogenesis.

## 1. Introduction

The cell cycle is divided into five phases, namely G0 (gap 0), G1, S (synthesis), G2, and M (mitosis). Genomic material is duplicated during the S phase, and it is distributed between newly formed daughter cells during mitosis [1]. Mitosis consists of distinct phases: prophase, prometaphase, metaphase, anaphase, and telophase, followed by cytokinesis [2,3]. Following the nuclear envelope breakdown at the prophase/prometaphase transition, a dynamic interaction is initiated between chromosomes and microtubules of the bipolar spindle, leading to chromosome bi-orientation and alignment at the metaphase plate, and subsequent segregation during anaphase. Cell cycle progression is controlled by the family of serine/threonine kinases called cyclin-dependent kinases (Cdk 1, 2, 4, and 6) and their regulatory subunits known as cyclins (A, B, D, and E), whereas chromosome segregation is monitored by the spindle assembly checkpoint (SAC) [4].

Spindle formation and disruption during mitosis lead to cell cycle arrest at the G2/M phase. Microtubule-targeting agents (MTAs), such as laulimalide, paclitaxel, docetaxel, cabazitaxel, and ixabepilone, stabilize the microtubule network [5,6], while colchicine, vinblastine, maytansine, and pironetin destabilize the microtubule network [7]. As a result, the polymerisation process is inhibited [8]. By perturbing spindle assembly, MTAs activate the SAC, which induces mitotic arrest and subsequent cell death. Cells may undergo apoptosis and die during mitosis or end mitosis without division, becoming tetraploids (4 N) in a process known as mitotic slippage [9].

However, microtubules not only form the mitotic spindle during cell division, but also maintain cell shape, intracellular trafficking, and cell motility [10]. Disrupting the microtubule dynamics impair all of these processes. Unfortunately, MTAs lack selectivity between malignant cells and normal cells, so many patients treated with MTAs experience neurotoxicity, which affects movement, sensation, and even organ dysfunction. Additionally, patients may experience a reduced production of platelets, erythrocytes, and leukocytes [11]. Although effective in various cancers, the use of MTAs throughout cancer therapy is currently limited due to their severe side effects. However, the concept of therapeutically inhibiting microtubule function during cancer cell mitosis remains compelling. The main challenge is to identify cell cycle regulators that are essential for the mitosis of cancer cells rather than normal cells.

Nowadays, SAC is at the heart of existing and emerging antimitotic interventions. Its activation requires the hierarchical recruitment of core SAC proteins, namely Bub1 (budding uninhibited by benomyl 1), Bub3, BubR1 (Bub1-related 1), Mad1 (mitotic arrest deficiency 1), Mad2, Mps1 (monopolar spindle 1), and Aurora B, to unattached kinetochores. The hypothesis that SAC activation or silencing could offer a realistic approach for killing cancer cells without affecting microtubules has led to the development of the so-called second-generation of antimitotics, which can be grouped into mitotic blockers (e.g., ispinesib, alisertib) or mitotic drivers [12]. Unfortunately, these second-generation antimitotics have failed in clinical trials due to disappointing clinical results and/or high toxicity [13].

Mitotic slippage is one of the primary mechanisms of resistance against antimitotic drugs [9]. Introducing new strategies to accelerate apoptosis could potentially lead to cell death before mitotic slippage occurs. Additionally, innovative approaches that delay mitotic slippage would prolong the time that cells spend in mitosis, thereby allowing apoptosis signals to accumulate. However, the most important objective is to achieve drug selectivity towards tumour cells over normal cells, in order to enhance the therapeutic window of antimitotic therapies. For instance, the development of drugs that specifically and efficiently target cancer cells while sparing normal cells should reduce toxicity.

**Scheme 1:**
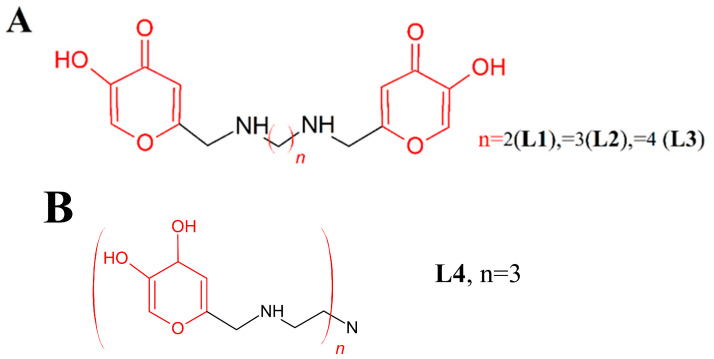
Molecular structures of the studied ligands (**A**) L1, L2, L3 and (**B**) L4 are depicted. Kojic acid units are highlighted in red.

Here, we introduce a novel small molecule L1 (Scheme 1A), which selectively inhibits mitosis solely in tumour cells. L1 is a derivative of kojic acid [14] and effectively binds essential metal ions (e.g., zinc(II), copper(II), iron(III)) [15]. In our previous studies, it was shown that L1 complexes with iron(III) ions bind to DNA [16], leading to inhibition of polymerase reaction. The antimitotic activity of L1 was incidentally observed during cellular studies on L1 cytotoxicity. The mechanism of mitosis inhibition by L1 is distinct from that of first- and second-generation antimitotics, leading to apoptosis.

In this study, we present our investigation of L1 action leading to tumour mitosis arrest in different cell lines through electron, fluorescence, and light microscopy, in addition to next generation sequencing (NGS) analysis. Furthermore, we used NGS, microscopy, fluorescence, and cytoflow analysis to examine the selective action of L1 in non-tumoural human kidney embryonic (HEK293T) and tumoural human epithelial colorectal adenocarcinoma (Caco2) cell lines. We also explored the bioavailability and cell toxicity of L1 in Caco2 and the malignant gliomas cell line (U118). Our findings show that L1 induces cell division arrest, ultimately leading to apoptosis within 24 h. Critically, the mitosis inhibition and apoptosis we observed in this study were limited to tumour cells.

## 2. Results

### 2.1. Investigating the L1 Mechanism of Action

Caco2 cells were cultured in cell culture media containing water solutions of L1, L2, or L3 molecules at a final concentration of 0.74 mM. Following 24 h of incubation, the cells’ morphology was evaluated using light microscopy (H&E staining). Only cells treated with the L1 ligand showed morphological changes characteristic of mitotic arrest. For this reason, further experiments were conducted only with the use of the L1 molecule.

Caco2 cells, both non-treated (control sample) and treated with the L1 ligand, were used to examine the cellular-level mechanism of L1 action. The morphology of the cells was observed using light and electron microscopy techniques, specifically scanning electron microscopy (SEM) and transmission electron microscopy (TEM), generating imaging at the micrometre and nanometre scales, respectively. Gene expression analysis was performed using next generation sequencing (NGS) on the non-treated Caco2 cells (control sample), cells treated with Colcemid (also known as Demecolcine, serving as the positive mitosis control [17]), and treated with L1.

#### 2.1.1. Light and Electron Microscopy Investigations

**Figure 1:**
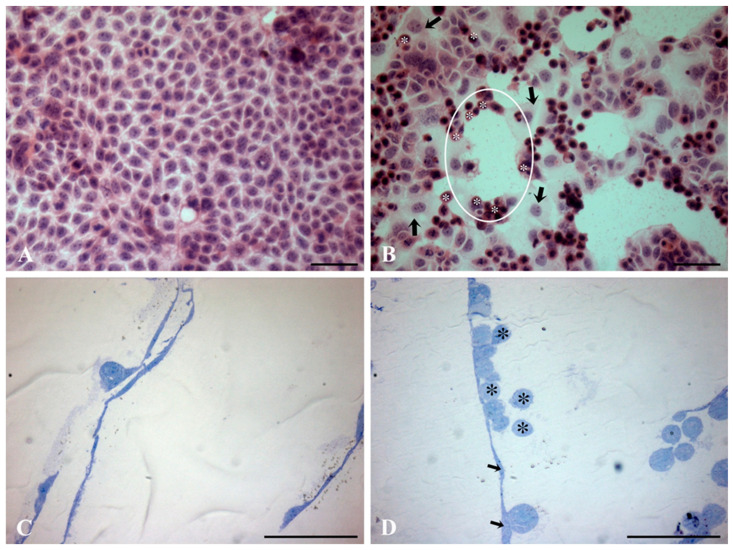
Light microscopic micrographs of both L1-treated (0.74 mM final concentration in the cell growing medium; 24 h) and control Caco2 samples using different light analysis procedures. (**A**,**B**) Hematoxylin and eosin (H&E) staining. (**C**,**D**) Sagittal sections of Caco2 cell culture embedded in resin, counterstained with toluidine blue. In the treated samples, H&E staining reveals the distinct distribution of mitotic cells (asterisks) arranged in circular “rosary-like” sequences (circle). Note the presence of cells in the interphase stage (arrows) (**B**). Sagittal sections (1 μm) of the resin-embedded L1-treated samples depict the distinct round shape of dividing cells (asterisks) positioned superficially to the flattened cells in the interphase stage (arrows) (**D**). Bars = 100 µm.

Preliminary light analysis using different staining methods on both L1-treated and control Caco2 samples revealed notable differences in their morphology. Both samples exhibited a well-developed cell monolayer. In the control sample, the cells were large, polygonal, and flattened with a large central nucleus, growing closely together in confluent areas. Most cells were observed in the interphase stage of the cell cycle, with only occasional observations of mitotic figures (Figure 1A). In contrast, Caco2 samples that were subjected to L1 treatment showed distinct morphological variations and different cell types at distinct stages in the cell cycle. The most prominent distinguishing feature in the L1-treated samples was the large number of dividing cells arrested at the mitotic stage (Figure 1B). Notably, H&E staining revealed the distinct distribution of mitotic cells forming circular “rosary-like” sequences (Figure 1B). Additionally, the 1 µm sagittal sections of the L1-treated cell culture embedded in resin highlighted the specific rounded shape of the dividing cells positioned superficially to the flattened cells in the interphase stage (Figure 1D).

**Figure 2:**
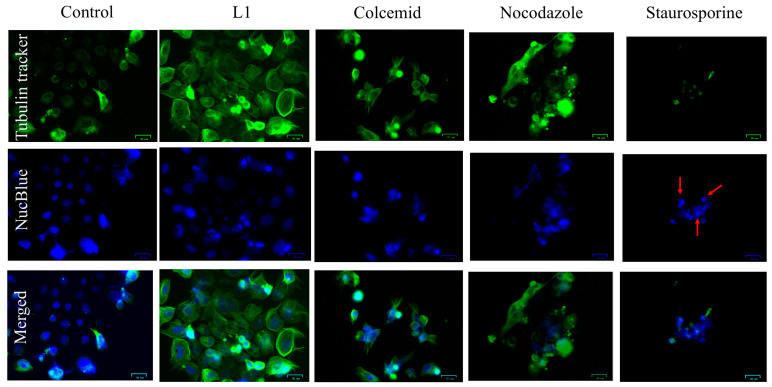
Live cell tubulin (green fluorescence) and nuclei visualisation (blue fluorescence) in Caco2 cells treated 24 h with L1 (0.74 mM; 228 µg/mL), Colcemid (130 nM), Nocodazole (66 nM), or Staurosporine (400 nM). In the NucBlue panel of the Staurosporine, the red arrows indicate a nucleus that is deteriorated to micronuclei. Bar = 30 µm.

The fluorescence staining of polymerized tubulin in live cells (Figure 2) was prepared for Caco2 cells growing 24 h with L1, Colcemid (binds to positive microtubule-end and suppresses microtubule dynamics), Nocodazole (interferes with the polymerisation of microtubules), and Staurosporine (apoptosis inducer). Staurosporine is a well-known apoptotic inducer, which exerts apoptotic events after a few hours. The 24 h treatment of Caco2 cells with Staurosporine led to late apoptotic stage in all treated cells with nucleus deterioration to micronuclei.

In the cell cultures treated with L1, Colcemid, and Nocodazole, numerous cells presented round-shape shrinked morphology and nuclear condensation (but retaining plasma membrane integrity) with intense green fluorescence of tubulin forming an apoptotic microtubule network (AMN). The microtubules were closely associated with the plasma membrane, forming a cortical ring or cellular “cocoon”.

**Figure 3:**
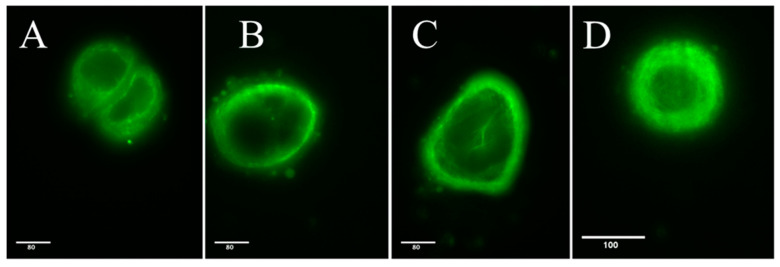
Fluorescence microscopy of live cell tubule staining in control (**A**) and L1-treated (**B**–**D**) Caco2 cells (24 h treatment, 0.74 mM). Bars = 80 and 100 μm, respectively.

The high-resolution live cell fluorescence staining (Figure 3) of L1-treated Caco2 cells showed that the AMN was formed beneath the plasma membrane, creating a ring or cortical “cocoon-like” structure that encloses most of the cell interior.

**Figure 4:**
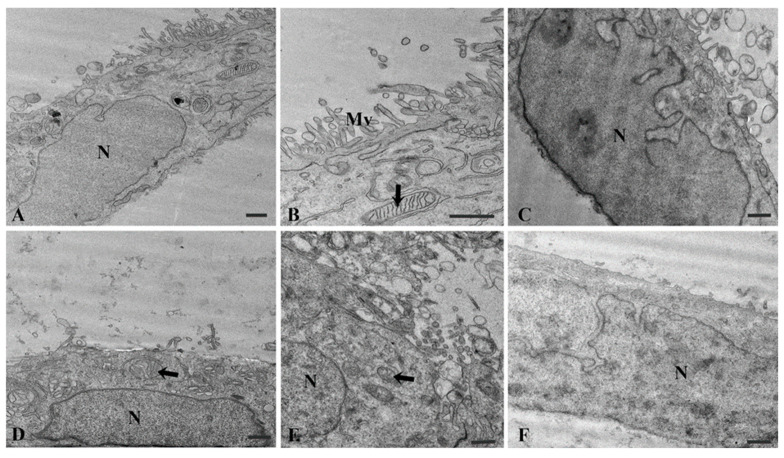
Electron micrographs of control Caco2 samples (**A**–**C**) and L1-treated (0.74 mM final concentration in the cell growing medium; 24 h) samples (**D**–**F**). The cellular ultrastructure of Caco2 cells in the interphase stage appears well preserved in both control and treated samples. Mitochondria (arrows). N = nucleus, Mv = microvilli. Bars = 1 µm.

The electron microscopic studies further confirmed and expanded upon the observations made using light microscopy. The transmission electron microscopy analysis revealed well-preserved cellular ultrastructure of all the examined Caco2 samples. Both L1-treated and control Caco2 cultures showed cells in the interphase stage with a well-preserved cellular ultrastructure, sharing similar morphological features and no apparent differences in their cell organisation. Specifically, the cells showed a continuous plasma membrane, which often displayed an irregular profile in the apical portion, where microvilli and undulating folds protruded towards the extracellular environment. These folds often appeared as short microvilli or exhibited a labyrinthine pattern, resulting in a significant amplification of the cellular surface. The cytoplasmic compartment in both samples showed a well-developed endoplasmic reticulum, Golgi apparatus, and numerous mitochondria with the typical internal organisation of foliate cristae. The nucleus typically exhibited a convoluted shape and appeared enclosed by a well-defined nuclear envelope, consisting of inner and outer membranes surrounding the perinuclear space. Most nuclei displayed prominent nucleoli and exhibited an euchromatin pattern in their chromatin (Figure 4).

**Figure 5:**
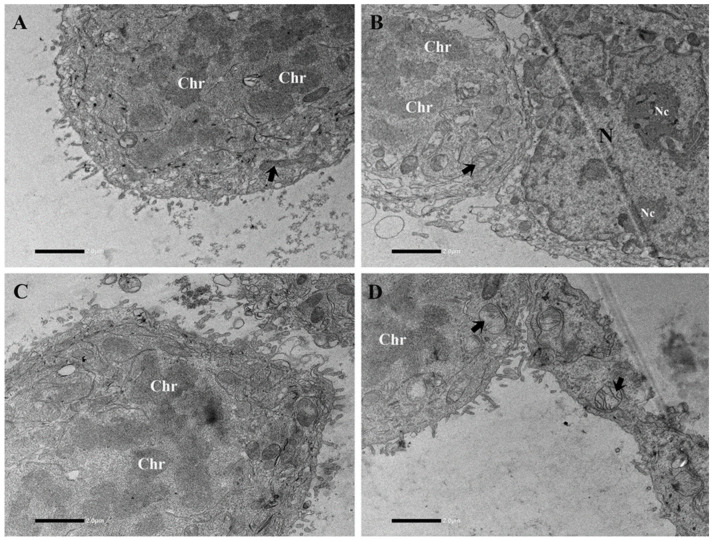
Electron micrographs of L1-treated (0.74 mM final concentration in the cell growing medium; 24 h) Caco-2 sample (different sections of the field are showed from (**A**–**D**)). Note in mitotic cells the loss of the nuclear envelope, whereas the adjacent cell in interface stage is characterized by the presence of the nucleus limited by a well-defined nuclear envelope (**B**). Most of cellular volume in mitotic cells is occupied by the presence of variously oriented chromosomes. Mitochondria (arrows). Chr = chromosomes, N = nucleus, Nc = nucleolus. Bars = 2 µm.

TEM analysis of the L1-treated samples confirmed the presence of numerous mitotic cells intermingled with cells in the interphase stage (Figure 5). These cells were round in shape and showed a loss in their nuclear envelope. Most of their cellular volume was occupied by chromosomes in various orientations, along with scattered mitochondria and other cytoplasmic organelles.

**Figure 6:**
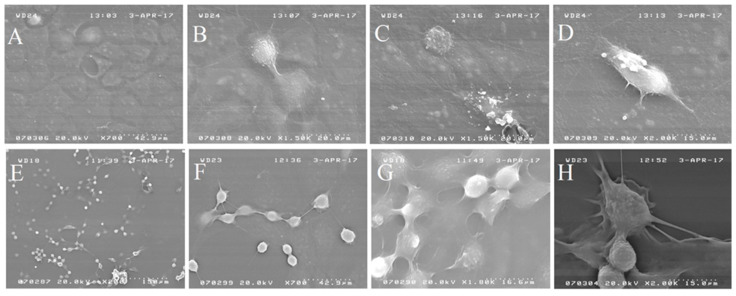
SEM images of (**A**–**D**) non-treated Caco2 cells. (**E**–**H**) Caco2 cells treated with L1 (0.74 mM final concentration in the cell growing medium; 24 h).

Scanning microscopic analysis (Figure 6) yielded well-preserved cells in the Caco2 control samples, which formed a monolayer (Figure 6A) with confluent cell areas. Occasionally, round-shape cells in the mitotic cell phase were observed (Figure 6B–D) and rare apoptotic zones.

Caco2 cells treated for 24 h with the L1 molecule (Figure 6E–H) showed significantly different morphology respect to the non-treated cells. The cells, less numerous respects to the non-treated cultures, mostly were round-shaped and seemed to maintain a close position forming discrete clusters of about 10–20 cells connected to each other by cytoplasmic extensions.

#### 2.1.2. Next Generation Sequencing (NGS) Investigation

**Figure 7:**
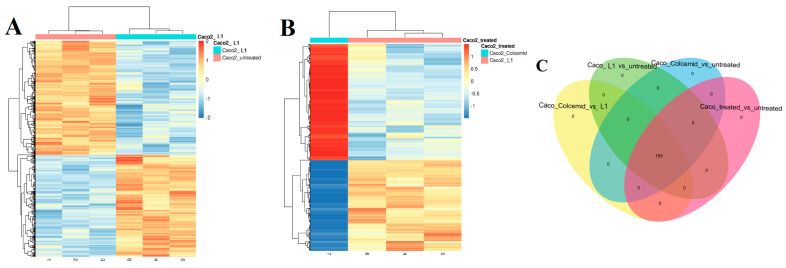
Heatmap of differentially expressed (DE) genes in (**A**) non-treated Caco2 cells and cells treated with L1 (0.74 mM final concentration in the cell growing medium; 24 h); (**B**) Caco2 cells treated with Colcemid and L1. Venn diagram (**C**) of DE analysis results with non-treated Caco2 cells and cells treated with L1 and Colcemid.

The NGS analysis was applied to investigate the differences in gene expression patterns between non-treated Caco2 cells (control sample) and cells treated with L1 for 24 h (Figure 7). The L1 concentration (0.75 mM) used for the NGS analysis was as low as possible to observe the mitotic arrest in most of the cells in the culture, and early-stage apoptosis after 24 h treatment. Our objective was to investigate the initial processes activated by the L1 treatment, rather than gene expression products of advanced apoptosis events. Moreover, the NGS analysis was carried out on the Caco2 cells treated with Colcemid for 24 h (Figure 7B), a compound that inhibits spindle formation and induces metaphase arrest during mitosis.

The heatmap analysis of DE genes showed substantial differences in gene expression between non-treated Caco2 cells and cells treated with L1 (Figure 7A). Additionally, the gene expression patterns in cells treated with Colcemid were significantly different than those treated with L1 (Figure 7B). The Venn diagram of DE genes in non-treated Caco2 cells and those treated with L1 or Colcemid showed the presence of 195 modulated genes (Figure 7C). However, an opposite gene modulation is observed for a subgroup of genes in the cells treated with Colcemid and L1 (the complete DE analysis is available in electronic Supplementary Materials).

Table 1 displays the 10 top DE genes affected by L1 treatment compared to non-treated cells. The comparative analysis of Caco2 cell genes affected by the treatment with Colcemid and L1 is presented in Table 2. The expression of tubulin beta (TUBB*) and tubulin alpha (TUBA*) genes (Table 2) was predominantly perturbed by Colcemid, whereas L1 treatment influenced the expression of the tubulin beta 2B class (TUBB2B) gene. Other genes affected by L1 treatment include INSIG1, IDI1, PNRC1, HERPUD1, and TP53INP2, among others (Table 1 and Table S1).

### 2.2. Investigation of Mitotic Exit into Apoptosis

Cells arrested at the G2/M phase may undergo apoptosis and die during mitosis or end mitosis without division. Thus, cells become tetraploids (4 N) in a process known as mitotic slippage. In order to investigate apoptotic events following cell cycle arrest, we examined Caco2 cells after 24 h treatment with L1 with the use of GFP CERTIFIED^®^ Apoptosis/Necrosis Detection Kit (catalogue Number: ENZ-51002). This kit labels early apoptotic cells with Annexin V-EnzoGold (enhanced Cyanine 3) and late apoptotic or necrotic cells with 7-AAD. Independent experiments were conducted for 24 and 48 h. The cells were treated with 0.74 mM L1 (concentration as low as possible to observe the mitotic arrest in most of cells in the culture), while Staurosporine treatment (4 μM) served as the positive control.

**Figure 8:**
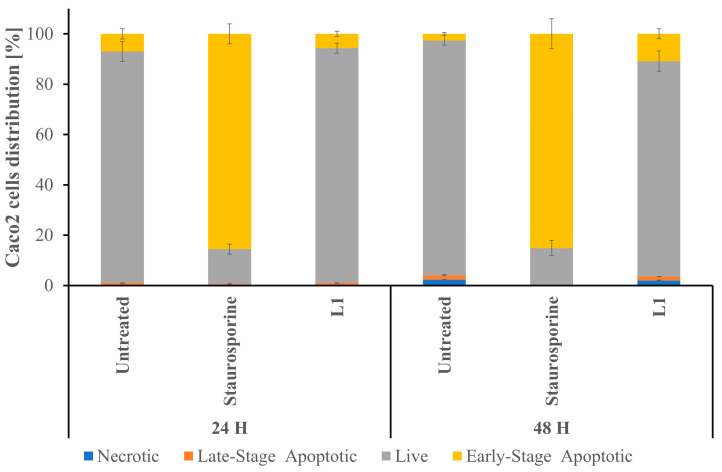
A graphical representation of Caco2 cell distribution across the four main populations (blue indicates necrosis; yellow indicates early apoptosis; orange indicates late apoptosis; and grey indicates live cells). The results were obtained after analysing dot plots, depicted in Figure S1. The data are presented as the mean ± SD (*n* = 3). Significant statistical differences between the control and samples were identified as *p* < 0.05.

Figure 8 illustrates the distribution of Caco2 cells among the four main populations, namely necrosis, late- and early-stage apoptosis, and live cells.

Following 24 h treatment with L1, the percentage of Caco2 cells in the early apoptotic stage remained comparable to the non-treated cells, as well as the percentage of late apoptotic and necrotic cells remained comparable (see Figure 8 and Figure S1). After 48 h of L1 treatment, the percentage of early-stage apoptosis cells doubled (with respect to the samples treated with L1 for 24 h), while the percentage of early apoptosis cells in the control sample lowered (with respect to the control samples growing for 24 h). The 24 h treatment with Staurosporine led to the apoptosis development in most of the growing cells and remained invariable after 48 h treatment.

The live cell imagining of Caco2 cells after 24 h treatment with L1 and Staurosporine (Figure S2) confirms the presence of rare necrotic events in the cell population, occurring mostly in the overpopulated areas.

**Figure 9:**
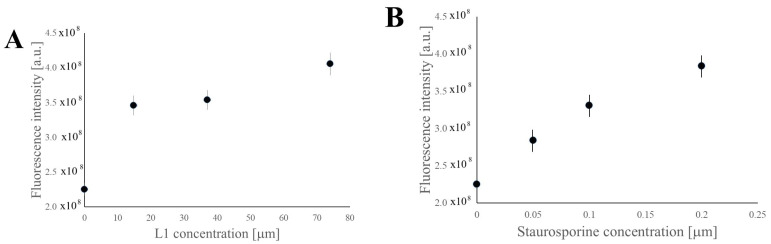
Caspase-3 quantitative analysis of Caco2 cell lysate after 24 h treatment with growing concentrations of L1 (**A**) and 5 h treatment with growing concentrations of Staurosporine (**B**). The data are presented as the mean ± SD (*n* = 3). Significant statistical differences between control samples (concentration = 0 [µM]) and experimental samples were identified as *p* < 0.05.

The Caspase-3 Activity Assay Kit (Manufacturer: Cell Signaling Technology, part number of the product:#5723, EuroClone, Via Figino 20/22, Pero (Milan) 20016, Italy) was used for the quantitative analysis of Caspase-3 concentration in the Caco2 cell lysate after 24 h treatment with growing concentration of L1 (Figure 9A) and 5 h treatment with increasing concentrations of Staurosporine (Figure 9B) as a positive control. Because Caspase-7 shares the same substrate sequence as Caspase-3, this kit also detects Caspase-7 activity. Figure 9 shows that both L1 and Staurosporine led to the significant increase in Caspase-3 and/or Caspase-7 in the cell lysates of Caco2 cells.

In addition, Caspase-3 and/or Caspase-7 was quantified in the HEK293T cell lysates after 24 h treatment with a growing concentration of L1 (Figure S3A) and 5 h treatment with increasing concentrations of Staurosporine (Figure S3B) as a positive control. There was no increase of caspases concentration in the HEK293T samples treated for 24 h with L1, while there was a significant growth of caspases concentration in the samples treated for 5 h with Staurosporine above 5 [µM] concentration.

### 2.3. Studies of L1 Selectivity Versus Tumour Cells

#### 2.3.1. Light Microscopy Investigation of L1 Selectivity

**Figure 10:**
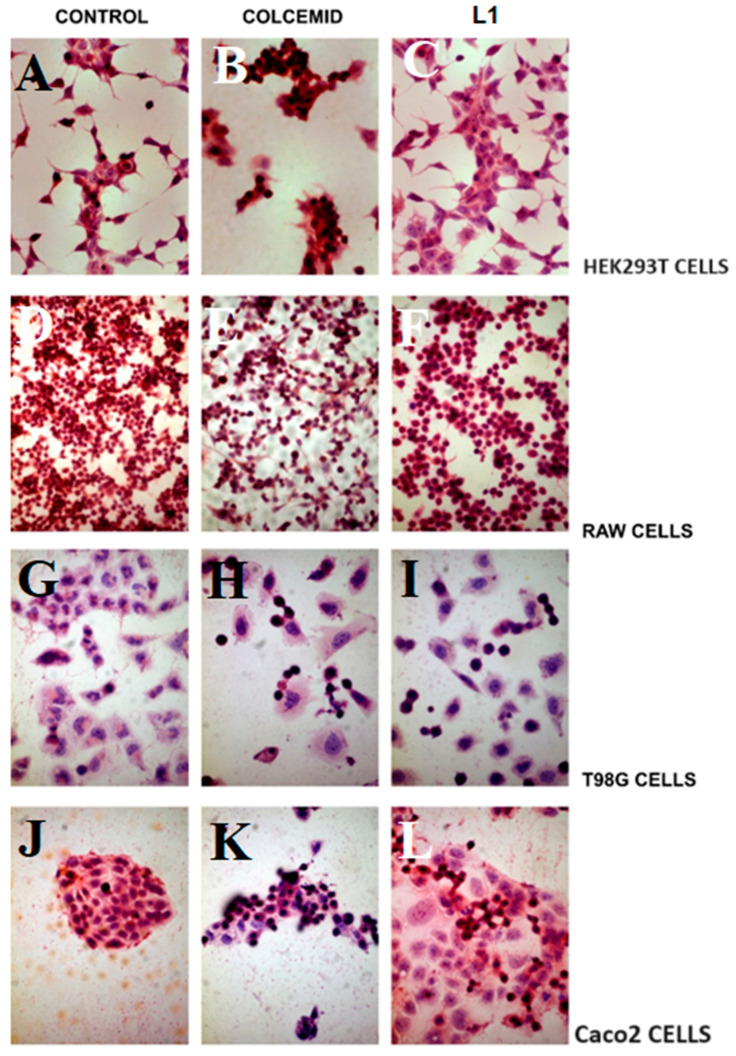
H&E staining of non-tumoural and two tumoural cell lines observed at ×10, ×20, and ×40 magnifications (**A**–**C**) HEK293T, (**D**–**F**) RAW, (**G**–**I**) T98G, and (**J**–**L**) Caco2 cell lines. Panels (**A**,**B**,**G**,**J**) depict non-treated cells (control samples). Panels (**B**,**E**,**H**,**K**) show the Colcemid-treated cells (positive control samples), while the panels (**C**,**F**,**I**,**N**) show cells treated with L1 (0.74 mM) for 24 h.

The mitotic morphology of various cells, including round-shaped cells detaching from the cell growing surface, can be easily observed under light microscopy. To investigate the selectivity of L1 against tumour cell lines, both non-tumoural cell lines (HEK293T and RAW) and tumoural cell lines (Caco2 and T98G) were cultured in medium containing L1 or Colcemid (positive control) for 24 h. The HEK293T and RWA cell lines are a well-established model of non-tumoural cells and are widely used in the studies of selective anticancer activities of pharmaceutics [18,19,20,21,22,23]. Following treatment, the samples were stained with H&E and the results are presented in Figure 10.

All cell lines cultured with Colcemid (Figure 10B,E,H,K) exhibited the characteristic morphology of mitosis arrest. However, only the tumoural cell lines (Caco2 and T98G) grown in the medium containing L1 (Figure 10I,L) showed the morphology indicative of mitotic arrest. The non-tumoural cell lines (HEK293T and RAW) cultured with L1 (Figure 10C,F) displayed cell morphology similar to that observed in control samples (Figure 10A,D).

#### 2.3.2. Investigation of L1 Selectivity Using Next Generation Sequencing (NGS)

**Figure 11:**
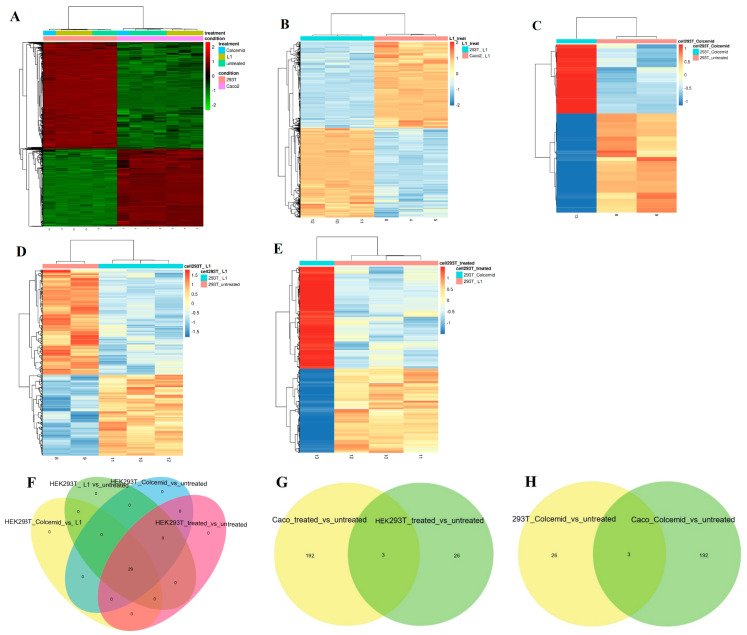
(**A**) Heatmap of the top 10% most variable genes. Heatmap of DE genes. (**B**) Caco2 and HEK293T cells treated with L1. (**C**) Non-treated and Colcemid-treated HEK293T cells (**D**) Non-treated and L1-treated HEK293T cells, and (**E**) Colcemid- and L1-treated HEKHEK293T cells. (**F**) Venn diagram of DE analysis results of non-treated HEK293T cells and cells treated with L1 or Colcemid. (**G**) Venn diagram of DE analysis results of Caco2 and HEK293T cells treated with L1. (**H**) Venn diagram of DE analysis results of Caco2 and HEK293T cells treated with Colcemid.

To assess L1 selectivity, we examined an NGS non-tumoural cell line (HEK293T) and a tumoural cell line (Caco2) after growing for 24 *h* in a medium containing L1 (0.75 mM) or Colcemid. The results of this comparative NGS analysis demonstrated the selective mitosis inhibition by L1 in the tumoural cell line (Caco2), whereas the cultures in Colcemid exhibited effective but non-selective mitosis inhibition, as shown in Figure 11 and Table 3 and Table 4.

It is evident that Caco2 and HEK293T cells treated with L1 showed a significantly different gene expression pattern (Figure 11). However, the gene expression pattern of HEK293T cells treated with L1 was not significantly perturbed compared to non-treated HEK293T cells (Figure 11D and Table 4).

The L1 treatment activated distinct gene expression profiles in Caco2 and in HEK293T cell lines. Indeed, the comparative gene analysis between HEK293T cells treated with L1 and Caco2 cells treated with L1 (Figure 11B) revealed a significantly different pattern. Furthermore, the gene expression patterns of HEK293T cells growing in the presence of L1 exhibited distinct gene expression profiles. The presence of Colcemid in the cell growing medium of HEK293T cells perturbed the expression of tubulin-related genes, namely TUBA1B, TUBB, TUBB4B, and TUBA1A (Table 3 and Table 4), exactly as in Caco2 cells (Table 2), thus confirming lack of the selectivity.

The Venn diagram of DE genes in HEK293T cells non-treated or treated with L1 or Colcemid showed that there are only 29 genes modulated in the same manner. Of note, the Venn diagrams of Caco2 and HEK293T cells treated with L1 (Figure 11G), and the Venn diagram of Caco2 and HEK293T cells treated with Colcemid (Figure 11H), showed the presence of three genes upregulated in Caco2 and HEK293T cells lines by both treatments. Those three genes, namely ENSG00000233822.6 (H2BC15, H2BFD, HIST1H2BN), ENSG00000184678.11 (H2B, H2B-GL105, H2B.1, H2B/Q, H2BE, H2BFQ, HIST2H2BE), and ENSG00000274750.3 (H3.1, H3/D, H3FD, HIST1H3E), encode histone proteins.

#### 2.3.3. Investigation of Selective Cell Cycle Arrest Using Fluorescence

To study the cell cycle phase in non-tumoural (HEK293T) and tumoural (Caco2) cell lines growing in the medium containing L1 for 24 h, we used a NUCLEAR-ID^®^ Red Cell Cycle Kit (GFP CERTIFIED^®^; EuroClone Via Figino 20/22 Pero (Milan) 20016, Italy). This kit offers a convenient approach for studying the induction and inhibition of cell cycle progression through flow cytometry and live cell imaging. In flow-cytometry quantitative analysis, it enables the percentage of cells in G0/G1, S, and G2/M phases to be determined, as well as the quantification of cells in the sub-G1 phase. The NUCLEAR-ID^®^ Red DNA stain can permeate the membrane of live cells and specifically target double-stranded DNA (dsDNA). Additionally, a control cell cycle perturbation agent, Nocodazole [24], was used as a positive control to monitor changes in cell cycle dynamics.

**Figure 12:**
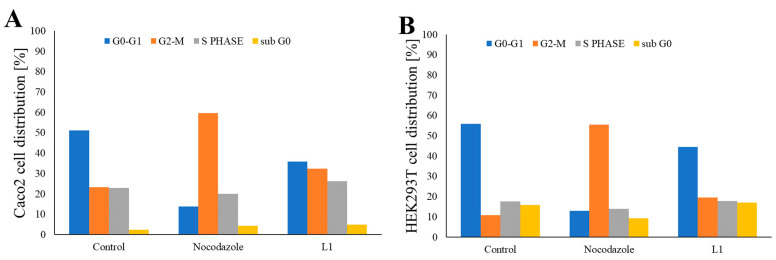
Graphical representation of the distribution of (**A**) Caco2 and (**B**) HEK293T cells in the G0-G1 (blue), S (grey), G2-M (orange), and sub G0 (yellow) phases of the cell cycle following growth in the non-treated medium, the medium containing Nocodazole, or the medium containing L1. Caco2 and HEK293T cells were treated using 0.66 μM Nocodazole and 0.74 mM L1 for 16 and 24 h, respectively. The results were obtained through the analysis of Gaussian plots, as depicted in Figure S4. The data are presented as mean ± SD (*n* = 3). Significant statistical differences were observed between the control and sample groups, where *p* < 0.05.

The results of the Cytoflow fluorescence investigation, which evaluated cell cycle arrest in different cell cycle phases, are presented in Figure 12. The mitotic indexes are presented in Table S3.

Caco2 cells grown in the normal cell growing medium were predominantly in the G0-G1 phase (accounting for approximately 50% of the cells), whereas the remaining cells were distributed across the S phase (~21% of cells) and the G2-M phase (~26% of cells). Caco2 cells treated with Nocodazole were primarily in the G2-M phase (accounting for approximately 60% of cells). L1 treatment led to the decrease of the cells in the G0-G1 phase (~35% of cells), with contemporary increase of the cells in the G2-M phase (~32% of cells) and S phase (~26% of cells).

Similarly, HEK293T cells cultivated in the normal cell growing medium were primarily in the G0-G1 phase (accounting for approximately 50% of cells). Cells in the S phase accounted for approximately 17%, whereas cells in the G2-M phase represented approximately ~16% of the total cell population. Treatment with Nocodazole induced cell cycle arrest predominately in the G2-M phase (accounting for approximately 56% of cells), confirming the lack of Nocodazole selectivity. Interestingly, L1 treatment did not significantly alter the distribution of cells in three cycle phases in the HEK293T cell population, with approximately 46% of cells in the G0-G1 phase, 17% of cells in the S phase, and 23% of cells in the G2-M phase.

**Figure 13:**
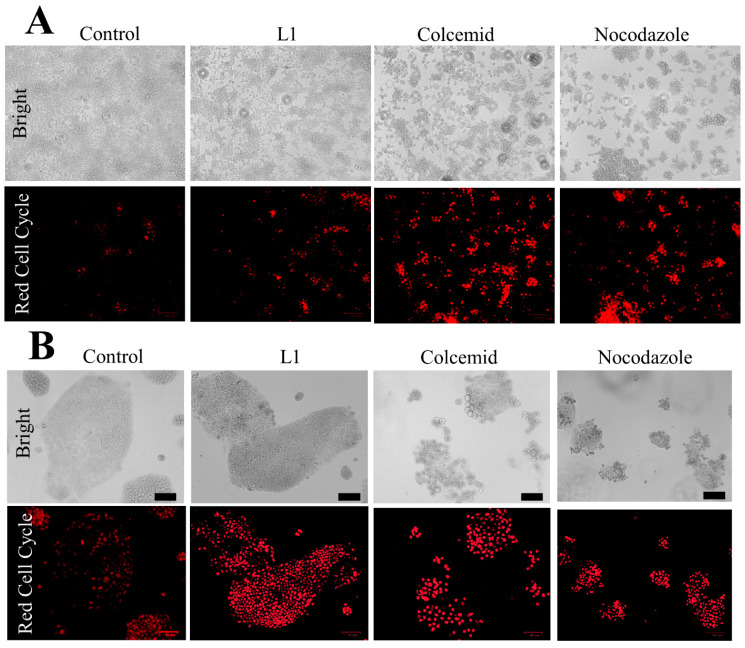
Red cell cycle visualisation in HEK293T cells (**A**) and Caco2 cells (**B**) treated with L1 (0.74 mM mg/mL), Colcemid (130 nM), or Nocodazole (66 nM). Bar = 70 µm.

In addition, the qualitative live-cell red cell cycle staining was prepared with Caco2 and HEK293T cells treated 24 h with L1, Colcemid, or Nocodazole as the positive control of cell cycle arrest (Figure 13). The intense red fluorescence appears in the cells with elevated concentration of dsDNA. Thus, both Caco2 and HEK293T cells treated with Colcemid or Nocodazole showed intense fluorescence staining. The treatment with L1 led to the red fluorescence staining in Caco2 cells, while only a few HEK293T cells were stained.

### 2.4. Bioavailability and Toxicity Studies of L1 in Different Cell Lines

Two different cell lines (Caco2 and U118) were used to evaluate L1 absorption. The amount of L1 ligand absorbed within 24 *h* was measured using UV techniques. Both Caco2 (29 ± 6% of the initial 0.74 mM concentration) and U118 cells (24 ± 7% of the initial 0.74 mM concentration) absorbed a similar quantity of ligand. Additionally, the total cell number, viability percentage, and cell size were evaluated using Trypan Blue staining and the automated LunaFL system (Figure S5). The number of Caco2 cells treated with L1 for 24 h was four times lower than that of the control sample (Figure S5A), whereas the viability (Figure S5B) and cell size (Figure S5C) remained unchanged. In the case of U118 cells treated with the L1 ligand, the total cell number decreased five-fold compared to the control sample, whereas the cell size and viability only slightly decreased (Figure S5D–F).

In the next experiment, we analysed the total cell number and cell viability of Caco2 cells treated with L1 across different time durations: 2.5, 4.5, 24, and 48 h (Figure S6). Morphological changes in most cells were observed as early as 4.5 h after treatment. The cell viability remained consistently high and unchanged throughout all experiments (Figure S6B). However, the cell number significantly decreased after 24 h of treatment and remained low after 48 h.

In the following step, Caco2 cells were treated with various concentrations of the L1 ligand for 24 h. The occurrence of morphological changes and the number of affected cells depended on the L1 ligand concentration in the cell growing medium. No morphological changes were observed in solutions with L1 concentrations ranging from 0.017 to 0.035 mM. The minimum ligand concentration that induced morphological changes in a few cells was 0.069 mM. As the ligand concentration increased, the number of affected cells increased, and in the solution containing a ligand concentration of 2.22 mM, all cells exhibited modified morphology. At concentrations above 3.33 mM L1, the cells detached from the edges of the cell growing flask.

The MTT assay was used to calculate the IC50 value of L1 in non-tumoural HEK293T cells (IC50 = 7 mM) and tumoural Caco2 (IC50 = 68.2 mM), SW480 (IC50 = 15.5 mM), and HT29 (IC50 = 4.7 mM) cell lines.

The dose-response analysis with the use of cell cycle phase investigation (Figure S7) showed that 0.45 mM concentration of L1 is necessary to arrest half of the cell culture population in the G2-M phase.

## 3. Discussion

Mitosis is a cell division process in which genetic material is duplicated and distributed between two newly formed daughter cells. One of the most distinctive features of mitosis, which can be easily observed through microscopic examination at the cellular level, is chromatin condensation [25]. Additionally, during mitosis, cells undergo a characteristic roundup phase and exhibit an intensely microvillous surface prior to cytokinesis [26]. Hematoxylin and eosin (H&E) staining is a technique that allows for rapid, qualitative analysis of the entire cell population. Hematoxylin stains cell nucleic acids within the nuclei (appearing purplish-blue), whereas eosin stains the extracellular matrix and cytoplasm proteins (appearing pink). The light microscopy examination of Caco2 cells treated with L1 (Figure 1) clearly showed that nearly half of the cell population presented mitotic morphology. In contrast, the control cell population only showed sporadic mitotic cells. Electron and scanning electron microscopy images (Figure 4, Figure 5 and Figure 6) confirmed the light microscopy observations, highlighting at ultrastructural level the morphological features that characterize the mitotic arrest event following L1 treatment.

The cytoflow studies using fluorescent markers to identify cell cycle phases showed a ~10% increase in the proportion of Caco2 cells in the G2-M phase (Figure 12A) following a 24 h treatment with L1 compared to Caco2 cells grown under normal experimental conditions. Notably, L1 showed selectivity versus non-tumoural HEK293T, which was not observed for Nocodazole.

Tubulin, a heterodimer composed of α and β subunits, exists in various isotope forms of differing primary sequences, and is encoded by different genes. To date, six α-tubulin and seven β-tubulin isotypes (encoded by multiple genes and exhibiting various tissue expression) have been identified. The β-isotypes differ only in the last 15–20 amino acids at the carboxyl terminal of the β-chain tubulin [27]. Although βI-tubulin is constitutively expressed in many tissues, other β-isotypes show selective expression patterns. For example, βII-tubulin is highly expressed in the brain, βIII-tubulin is found in neurons and Sertoli cells, and βIVa and βIVb are predominantly expressed in the brain and various tissues, respectively [28]. Tubulin is primarily located in the cytosol; however, certain β-tubulin isotypes have been detected in the nuclei of both normal and tumour cells [29]. Several studies have demonstrated that tubulin can exist in non-microtubule forms within the cells [30,31,32]. This is especially true for the βII isotype, which is often observed in cell nuclei, potentially forming a reticulum rather than microtubules [33]. Nuclear βII is highly abundant in cancer cells, less prevalent in cultured cells, and only minimally present in normal cells in situ [29,34,35]. Specific tubulin isotypes have been associated with certain forms of cancer. Previous research has shown that βII-tubulin is expressed in various transformed cells, predominantly in cell nuclei in a non-microtubule form [36]. βII-tubulin has been used for differentiating skin tumours and predicting treatment response in prostate cancer [37,38]. In colorectal cancer (CRC), tubulin expression has been correlated with the stage of the disease [39,40], and the analysis of CRC cell lines has revealed high expression of βI, βIVa, and βIVb, with only minimal amounts of βIII [41].

In a recent study conducted by Ruksha et al. [36], a cohort of 124 CRC patients was examined, revealing a significant association between the over-expression of βII and a significantly diminished life expectancy. Patients with nuclear βII exhibited an even shorter life expectancy. The authors postulated that βII-tubulin may contribute to cancer growth and metastasis, irrespective of its microtubule form.

In another study by Bouras et al. [42], antibodies against β-tubulin were used to examine patients with CRC and other gastrointestinal malignancies. The study included 54 CRC patients, 16 patients with gastric cancer, 10 patients with esophageal cancer, 10 patients with pancreatic cancer, 28 healthy subjects, and 6 patients with other colon-related conditions. The findings of this study demonstrated that β-tubulin serves as a cancer-specific antigen in a proportion of the cancer patients. Specifically, they identified β-tubulin as a cancer-related antigen capable of eliciting an immune response in patients with colorectal cancer and other gastrointestinal malignancies.

In our studies, through NGS analysis of both L1-treated and untreated Caco2 cells, we observed that among the top 10 genes most affected by L1 treatment, only one tubulin gene, namely tubulin beta 2B class IIb (TUBB2B; Table 1), exhibited significant changes. Conversely, the treatment of Caco2 cells with Colcemid (Table 2) led to a negative log2 fold change in the expression of both α and β tubulin genes, namely TUBB, TUBA1B, TUBB4B, and TUBA1C.

Another gene highly influenced by the 24 h treatment with L1 is insulin-induced gene 1 (INSIG1). This gene encodes an endoplasmic reticulum-associated protein that, together with sterol regulatory element-binding protein and cleavage-activating protein, downregulates cholesterol synthesis by inhibiting proteolytic processing of sterol regulatory element-binding proteins [43]. Previous studies demonstrated that INSIG2 serves as a positive prognostic biomarker for colon and pancreatic cancer. Sun et al. [27] conducted studies using samples derived from 78 human colon cancer specimens and showed that INSIG2 is a gene with univariate-negative prognostic capacity, capable of discriminating human colon cancer survivorship. Gain- and loss-of-function studies further revealed that the INSIG2 gene product is involved in cancer migration, invasion, and maintenance of the mesenchymal phenotype in vitro, and metastasis in vivo.

Research conducted by Kaneda et al. [44], using gastric cancer cell lines, showed that INSIG1 expression was markedly reduced in 19 cancers, including the 11 cancers with methylation. Although the decreased INSIG1 expression in cancers correlated with methylation at the edge of CpG islands in the promoter region, the methylation was likely a secondary change. The 24 h treatment of Caco2 cells with L1 led to a log2 fold change of 1.3 in the expression of the INSIG1 gene. The upregulation of gene expression observed during L1 treatment has the potential to restore normal gene expression in tumour cells.

The expression of isopentenyl-diphosphate delta isomerase (IDI-1) is also upregulated by L1 treatment in the Caco2 cell line. IDI-1 is a metal-dependent enzyme that catalyzes the isomerisation between isopentenyl diphosphate (IPP) and dimethylallyl pyrophosphate (DMAPP) in the final step of the mevalonate pathway. IPP and DMAPP serve as precursors for various terpenoids, including cholesterol. The expression of IDI-1 in cancer cell lines remains poorly understood. Interestingly, Suberoylanilide hydroxamic acid (SAHA) [45], a histone deacetylase inhibitor, exhibits high potency in inducing cell differentiation of cultured murine erythroleukemia cells. Its selective cytotoxic effects in breast cancer cells are manifested by G1 and G2/M cell cycle arrest and eventual apoptosis. SAHA modulates cell cycle and apoptosis regulatory proteins and induces several genes associated with differentiation and/or growth inhibition, including IDI1.

A set of genes, including PNRC1, HERPUD1, TP53INP2, Tob1, and SAT1, were downregulated in patients with different cancer types, and their decreased expression was correlated with poorer prognosis and shorter survival time. In Caco2 cells treated with L1 for 24 h, the expression of these genes is positively regulated.

The synthesis of ribosomes plays a central role in cell cycle progression and cancer proliferation. A recent study by Gaviraghi et al. [46] demonstrated that proline-rich nuclear receptor coactivator 1 (PNRC1), an mRNA decapping coactivator, regulates ribosome biogenesis and functions as a tumour suppressor. PNRC1 relocates the Dcp1/Dcp2 mRNA decapping complex to the nucleolus, promoting the decapping of specific snoRNAs to disrupt ribosomal RNA processing. By slowing down rRNA processing and thus ribosome biogenesis, PNRC1 acts as a gatekeeper, limiting oncogenic potential [47]. The treatment of Caco2 cell with L1 leads to 24 h results in an upregulation of PNRC1 gene expression (log2 fold change = 0.93).

Homocysteine-inducible ER protein with ubiquitin-like domain 1 (HERPUD1) is an ER membrane protein that is involved in the translocation of misfolded proteins from the ER to the cytosol. This translocation allows for direct 26S proteasome-mediated degradation and subsequent elimination of misfolded proteins [48,49]. Studies conducted on patient samples have determined that HERPUD1 levels were significantly suppressed in cancerous tissues compared to healthy tissues. The authors proposed that HERPUD1 may function as a tumour suppressor, inhibiting tumourigenesis [50], and could serve as a promising molecular target for new and effective anticancer therapy [51]. Another study has demonstrated that apoptotic cell death was induced in prostate cells that overexpressed HERPUD1 [52]. According to the NGS analysis of Caco2 cells treated 24 h with L1, there is HERPUD1 gene upregulation (Table 1).

Tumour protein 53-induced nuclear protein 1 (TP53INP1) is a stress-induced p53-target gene whose expression is modulated by transcription factors, such as p53, E2F1, and p73. Together with homeodomain interacting protein kinase-2 (HIPK2), TP53INP1 phosphorylates the p53 protein at Serine-46, enhancing its stability and transcriptional activity. This ultimately leads to the transcriptional activation of p53-target genes such as p21 and PIG3, resulting in cell growth arrest and apoptosis upon DNA damage stress [53]. TP53INP2 is crucial in gene transcription regulation and starvation-induced autophagy. Moreover, TP53INP2 is involved in DNA replication, DNA repair, cell cycle, and several metabolic pathways. Additionally, TP53INP2 can influence the expression of multiple genes by enhancing the transcriptional activity of nuclear hormone receptors. Ivanova et al. [54] demonstrated that TP53INP2 sensitizes cancer cells apoptosis induced by death receptors by acting as a scaffold for efficient ubiquitination of caspase-8 by TNF receptor-associated factor 6 (TRAF6). In a recent study, Cao et al. [55] observed that lower TP53INP2 expression in head and neck squamous cell carcinoma (HNSCC) patients compared to normal controls, whereas patients with higher TP53INP2 expression exhibited longer survival times. Hu et al. [56] conducted studies using the human liposarcoma cell line SW872 and found a low level of basal autophagy in SW872-S cells with high malignancy. They also observed a decrease in TP53INP2 expression during the malignant progression of liposarcoma in both cell lines and primary tissues. L1 treatment for 24 h led to increased TP53INP2 gene expression (Table 1), which coincided with the early-stage apoptosis events, further confirmed by cytoflow analysis.

Another protein that is involved in the early events of apoptosis and whose expression is enhanced after 24 h of L1 treatment is transducer of ErbB-2.1 (Tob1). Tob1 belongs to the Tob/B cell translocation gene (BTG) family of proteins and, when overexpressed, it arrests the cell cycle at the G1 phase [57,58,59]. Tob1 interacts with the ErbB2 receptor and functions as a negative regulator of cell growth by inhibiting ErbB2-mediated cell signalling pathways [60]. Several studies have shown that Tob1 expression is suppressed in various cancers, including breast [61], pancreas [62], thyroid [63], and stomach [64] cancer. In a recent study by Kundu et al. [65], the overexpression of Tob1 reportedly led to apoptosis induction, with concurrent inhibition of the proliferation, migration, and invasion of gastric cancer cells. This process is partially mediated by the activation of Smad4 and p15 expression, as well as the downregulation of β-catenin and its target genes (e.g., cyclin D1, uPAR, and PPARδ).

c-Jun is a signal-transducing transcription factor of the AP-1 family, which is involved in cell cycle progression, cell transformation, and differentiation, and is linked to apoptosis [66]. For example, increased expression of the c-Jun gene alone is sufficient to induce apoptotic cell death in NIH 3T3 fibroblasts [66]. c-Jun is considered a proto-oncogene as it encodes a component of the mitogen-inducible immediate–early transcription factor AP-1 and has been implicated as a positive regulator of cell proliferation and progression from G1 to S phase [67]. Schreiber et al. [67] showed that c-Jun negatively regulates transcription of p53 by directly binding to a variant AP-1 site in the p53 promoter. Following a 24 h treatment with L1, the expression of the JUN gene significantly increased, with a log2 fold change of 1.2.

SAT1 (spermidine/spermine N1-acetyltransferase 1) is a rate-limiting enzyme involved in polyamine catabolism, crucial for converting spermidine and spermine back to putrescine. The SAT1 gene is also a transcription target of p53. Recently, Ou et al. [68] found that SAT1 activation induces lipid peroxidation and sensitizes cells to undergo ferroptosis in response to reactive oxygen species (ROS)-induced stress, leading to the suppression of tumour growth in xenograft tumour models. Notably, the authors observed that SAT1 expression is downregulated in human tumours, and CRISPRcas9-mediated knockout of SAT1 expression partially abolished p53-mediated ferroptosis.

Tribbles proteins are pseudo-kinases that possess a structure of protein kinases but do not phosphorylate substrates. They are involved in the differentiation of myeloid cells [69] and adipocytes [70]; therefore, their dysregulation can contribute to neoplasms and metabolic disruption. Furthermore, emerging evidence suggests that Tribbles may contribute to the development of solid tumours and therapeutic resistance [71]. Additionally, the TRIB1 gene is closely genetically linked to the c-MYC oncogene, which could make it relevant in the context of solid tumours. After only 24 h of L1 treatment, there is an increase in TRIB1 gene expression (log2 fold change = 1.1) in Caco2 cells.

The enrichment analysis of the top 10 DE gene clusters showed that L1 treatment of Caco2 cells led to positive regulation of genes involved in various transcription pathways, the metabolism and biosynthesis of lipids, lipoproteins, steroid, cholesterol, and adipogenesis (Table S1, data presented for -LOG10 = 10 value clusters). Additionally, L1 treatment positively affected pathways related to ferroptosis, the ATF-2 and Ap-1 transcription factor network, and the TGF-beta, IL-18, and VEGFA-VEGFR2 signalling pathways. Other genes are negatively regulated by L1 treatment (Table S2, data presented for -LOG10 = 10 value clusters), particularly those involved in cell cycle, mitosis, DNA synthesis and repair, and fundamental biochemical cycles (e.g., citric acid cycle, TCA cycle).

In normal cells, phosphatidylserine (PS) residues are typically located in the inner membrane of the cytoplasmic membrane. However, during apoptosis, these PS residues are translocated to the membrane surface and become externalised by the flippases and floppases enzymes. This PS translocation is driven by the Caspase-3 and Caspase-7 cleavage of XRP-8 [72], and generally considered an early event in apoptosis. Annexin-V is a specific PS-binding protein that can be used to detect apoptotic cells. Late apoptotic cells and necrotic cells lose their cell membrane integrity and become permeable to vital dyes such as 7-AAD (a DNA intercalator). By employing both Annexin and 7-AAD in cytoflow studies, it is possible to quantify the percentage of cells in early and late apoptosis, as well as necrosis phases. The treatment of Caco2 cells with L1 did not increase apoptotic events (both early and late) within 24 h; however, extending the treatment duration up to 48 h increased slightly the percentage of apoptotic cells (Figure 8). Notably, the quantitative analysis of Caspase-3 and/or Caspse-7 in Caco2 cell lysate (Figure 9A) showed that 24 h treatment with L1 led to the significant increase in caspases, thus suggesting the possible involvement of L1 in the caspase pathway of PS externalisation during the early stages of apoptosis.

The apoptotic microtubule network (AMN) formation takes place in different cell lines and under numerous apoptotic stimuli. The studies of Sanchez-Alcazar et al. [73] showed that AMN is associated with the plasma membrane (typically organized beneath the plasma membrane) and during apoptotic events form a cortical ring or cellular “cocoon”, which is necessary to preserve the plasma integrity. AMN is present in cells containing active caspases; however, the role of caspases in AMN formation is still unclear. The fluorescence microscopy analysis of tubulin staining showed that in Caco2 cells treated for 24 h with L1, there is a formation of AMN with the prevalence of round-shape cells. Povea-Cabello et al. [74] indicated that the morphological and molecular changes observed in Caco2 cells treated for 24 h with L1 could be defined as “slow” or round-shaped apoptosis events, with caspase activation, cell detachment, microtubules remodelling, and formation of a round-shaped AMN. This is in contrast to “fast” or irregular–shaped apoptosis with formation of an irregular-shaped AMN and apoptotic cell morphology, which frequently shows apoptotic membrane protrusions or microtubule spikes.

The most crucial aspect of drug therapy is its ability to selectively target specific cells. In the case of L1 treatment, it induced mitotic arrest and subsequent mitotic morphology exclusively in cancer cells (Caco2 and T98G). Conversely, non-tumoural cells (HEK293T and RAW) treated with L1 exhibited normal cell morphology (Figure 10). Colcemid and Nocodazole, the effective mitotic inhibitors, lack the ability to distinguish between target and normal cells, leading to the development of mitotic morphology in all cells treated with Colcemid (Figure 10) and cell cycle arrest in G2-M phase in cells treated with Nocodazole. The morphological investigation, supported by H&E staining analysis, is further corroborated by NGS studies of Caco2 and HEK293T cells treated with L1.

The NGS analysis of HEK293T cells treated with L1 showed no significant perturbations in gene expression (Table 4) compared to untreated cells. Additionally, the gene expression pattern of HEK293T cells differed from that of L1-treated Caco2 cells cultured in medium (Table 1). In contrast, the non-selective Colcemid treatment of HEK293T cells led to the downregulation of tubulin genes (TUBA1B, TUBB, TUBB4B, and TUBA1A; Table 3), similar to the observation in Caco2 cells treated with Colcemid (Table 2).

Finally, the fluorescent investigation of cell cycle phase (Figure 10B) demonstrated similar distribution of sub G0, G0-G1, S, and G2-M phases in both L1-treated and untreated HEK293T cells. Nocodazole, a known mitosis inhibitor, increased the percentage of cells in the G2-M phase in both in Caco2 (Figure 12A) and HEK293T (Figure 12B) cell populations.

The L1 molecule is highly soluble in water, enabling easy absorption by cells from the growth medium. An increase in mitotic arrest events can be observed as early as 4 h after treatment. Following a 24 h treatment with L1, a significant reduction in the total tumour cell count was observed, with the majority of remaining cells arrested in mitosis. This mitotic inhibition subsequently led to apoptosis, with early apoptotic events detected already after 24 h of treatment.

## 4. Materials and Methods

### 4.1. Reagents

The following reagents were used for the synthesis of the ligands without additional purification: 5-hydroxy-2-hydroxymethyl-pyran-4-one (kojic acid (KA); purity 99%), ethylenediamine, propane-1,3-diamine, butane-1,4-diamine, and tris(2-aminoethyl)amina, all of which are Aldrich products. Glutaraldehyde, (para)formaldehyde, sodium cacodylate, Embed 812 resin, osmium tetroxide, and bismuth subnitrate were procured from Electron Microscopy Sciences, Hatfield, PA, USA. Staurosporine, Nocodazole, and Colcemid were TCI Europe products.

### 4.2. Molecules Synthesis and Characterisation

The ligands were synthesised according to procedures published in previous research [15,75]. The data that describe the final products are available in Supplementary Materials.

### 4.3. Cell Cultures

The following cell lines were obtained from the Istituto Nazionale per la Ricerca sul Cancro c/o CBA (ICLC, Genova, Switzerland): commercial HEK293T human kidney embryonic cell line (catalogue code HTL03003), RAW mouse, BALB/c; macrophage (catalogue code ATL02001), and Caco2 Human Epithelial Colorectal Adenocarcinoma (catalogue code HTL97023). The T98G Glioblastoma Multiforme (catalogue code CRL-1690) and U118 malignant gliomas cell lines (catalogue code HTB-15™), SW480 human large intestine of a Dukes C colorectal cancer (catalogue code CCL-228™), and HT29 human epithelial colorectal adenocarcinoma (catalogue code HTB-38™) were obtained from the atcc.org. The identification of these cell lines was confirmed in January 2022 using short tandem repeat (STR) profiling. Multiplex PCR analysis was performed using human, rat, and mouse primers and subsequently confirmed as human using cytochrome c oxidase subunit I (cox I) primers. The cells were cultured for 24 h under different conditions before being assessed.

The cell culture medium comprised a mixture of minimal essential medium, MEM (EBSS), 10% fetal bovine serum (EuroClone, Via Figino 20/22, Pero (Milan) 20016, Italy), FBS South America origin EU approved—highly tested, cod. ECS5000L), 100 units per ml penicillin, 100 mg × mL streptomycin, 2 mM glutamine, and 1% non-essential amino acids. To achieve the desired experimental conditions, confluent cells were isolated using trypsin/EDTA, and cell samples of 2–3 × 10^4^ cells per cm^2^ were seeded in 35 mm dishes and incubated at 37 °C and 5% CO_2_. After 24 h of growth with the complete medium, the cells were subjected to various experimental conditions as described in Section 4.1, Section 4.2, Section 4.3 and Section 4.4.

### 4.4. Cytotoxic Activity

#### 4.4.1. Trypan Blue Staining

The cytotoxic effects of different molecules were quantitatively evaluated in the cells. Specifically, the total cell number, cell viability (%), and cell size were evaluated using the LunaFL Cell Counter (www.logosbio.com, accessed on 18 December 2024) according to the manufacturer’s user manual. To conduct the analysis, cell samples were mixed in a 1:1 ratio with a 0.2% Trypan Blue solution supplied in the reagent kit. Cells were counted in the Luna cell counting chamber slide within 1–3 min. The results are presented as the percentage of cell viability compared to non-treated control cells under the same experimental conditions. To ensure accurate measurements, the cell count was repeated at least three times, and at least three independent experiments were conducted under the same experimental conditions.

#### 4.4.2. MTT Assay

The HEK293T, Caco2, SW480, and HT29 cells were cultured for 24 h at 37 °C and CO_2_ (5%) at 1.4 × 10^4^ cells per well concentration. After 24 h growth, the cell culture medium was replaced with the cell culture medium containing increasing concentration of L1. Stock solution of L1 was prepared daily in the complete cell culture medium with 10% fetal bovine serum and sequentially diluted in the complete cell culture medium with 10% fetal bovine serum to obtain a concentration range of 0 (control sample)–20 mM (the highest L1 concentration, which does not perturb the cell culture medium pH). Next, 100 μL of these prepared compound solutions in cell culture medium were added in triplicates to a 96-well plate where the cells were growing. Successively, the plates were incubated for 24 h. MTT solution (10 μL) was prepared at a concentration of 5 mg mL^−1^ in Dulbecco’s phosphate buffered saline (DPBS) was added to the cells, and the plates were incubated for an additional 4 h. The culture media were carefully aspirated to preserve the purple formazan crystals that were dissolved in DMSO (100 μL per well). The absorbance of the resulting solutions, which is directly proportional to the number of surviving cells, was measured at 590 nm using the Thermo Scientific™ Multiskan™ SkyHigh (N. parte N21872; Thermo Fisher Scientific, 168 Third Avenue, Waltham, MA USA 02451) microplate reader, and the data were analysed with the free IC50 calculator available at https://www.aatbio.com/tools/ic50-calculator (accessed on 18 December 2024) using the four parameter mode. The reported IC50 values are based on the means of three independent replicates of the experiment, each comprising three tests per concentration level.

### 4.5. Determining L1 Bioavailability in the Cells

The bioavailability of the ligand was examined using the UV technique as described in previous studies [76]. Each ligand was introduced to the cell growth medium containing living cells and incubated for 24 h. The pH of the cell growth medium, both with and without cells, was carefully maintained over 24 h using a daily calibrated combined-glass electrode. The analytical determination of each ligand was performed at its maximum absorbance (316 nm) in the cell growth medium, and the calibration curve was generated using various concentrations of pure ligand in the medium to quantify the ligand. Each experiment was repeated at least three times in accordance with statistical analysis requirements.

### 4.6. Microscopy

Samples of both treated and non-treated control Caco2 cells were collected after 24 h of growth with complete medium, following procedures described in Section 2.3. These samples were then prepared and processed for light microscopy analysis.

#### 4.6.1. Light Microscopy

##### Hematoxylin and Eosin (H&E) Staining

One group of treated and non-treated control samples of Caco2 cells was fixed in cold acetone for 10 min, following by air drying for 30 min. The samples were then counterstained using hematoxylin and eosin (H&E), observed, and photographed using an Olympus BH2 light microscope (Tokyo, Japan).

##### Fluorescence Microscopy and Tubulin Staining

The groups of treated and non-treated control samples of Caco2 and HEK293T cells were observed and photographed with a Zoe^®^ microscope for live cell imaging. Bright-field and variable fluorescence wavelengths were applied to samples (blue channel λEx 355 ± 40 nm λEm 433 ± 36; green channel λEx 480 ± 17 λEm 517 ± 23; red channel λEx 556 ± 20 λEm 615 ± 61).

The live cell tubulin staining was prepared with the use of Tubulin TrackerTMGreen (Invitrogen, Part number T34075, Waltham, MA, USA) according to the manufacturer’s instructions. In the ZOE^®^ microscopy image analysis, the NucBlueTM Live Cell Stain (Invitrogen, Part number R37605) was used for the nucleus staining according to the manufacturer’s instructions.

Moreover, for detail tubulin staining, the Caco2 cell samples were observed and photographed with Nanoimager ONI (https://oni.bio/nanoimager/?utm_campaign=General+Campaign&utm_source=adwords&utm_medium=ppc&utm_term=oni%20nanoimager&hsa_acc=7022038699&hsa_cam=761253325&hsa_ver=3&hsa_net=adwords&hsa_src=g&hsa_tgt=kwd-1202981648086&hsa_grp=144725156772&hsa_ad=613687392916&hsa_kw=oni%20nanoimager&hsa_mt=b&gad_source=1&gclid=CjwKCAjw88yxBhBWEiwA7cm6pRrsaGHqA5vjtU-LKpxfzYdbVLTGj184AvI_XlLaNFq2D7QcT1VL0RoCz5MQAvD_BwE, accessed on 18 December 2024).

##### Toluidine Blue Staining

A third group of treated and non-treated control samples of Caco2 cells was fixed in a solution of 1% (para)formaldehyde and 1.25% glutaraldehyde in 0.1 M sodium cacodylate buffer for 2 h. Samples were then embedded in Embed 812 resin and polymerized in an oven at 60 °C for 24 h. Finally, 1 μm thick sections were cut, stained with toluidine blue, observed, and photographed using an Olympus BH2light microscope.

#### 4.6.2. Electron Microscopy

##### Transmission Electron Microscopy (TEM)

Treated and non-treated control samples of Caco2 cells were fixed for 2 h in a solution of 1% (para)formaldehyde and 1.25% glutaraldehyde in 0.1 M sodium cacodylate buffer at pH 7.4. After rinsing in the same buffer, the samples were post-fixed in 1% osmium tetroxide for 1 h and stained overnight at 4 °C in aqueous uranyl acetate (0.25%). Dehydration of the cell cultures was performed using an ascending graded series of ethanol and xylene, and followed by infiltration and embedding in Embed 812 resin. The specimens were then transferred to flat embedding moulds and polymerized in an oven at 60 °C for 24 h. Finally, thin sections (60–90 nm thick) were obtained using a LKB ultratome 8800 ultramicrotome. The sections were post-stained with uranyl acetate and bismuth subnitrate, and observed and photographed in a transmission electron microscope (JEOL 1400 plus model, Tokyo, Japan) operating at 80 kV.

##### Scanning Electron Microscopy (SEM)

The Caco2 cells obtained as described above were fixed for 30 min in paraformaldehyde and 1.25% glutaraldehyde in 0.1 M sodium cacodylate buffer, pH 7.4, and then washed in PBS (phosphate buffered saline) and treated with a solution of 1% osmium and 1.25% ferrocyanide for 1 h in a humid chamber.

After numerous washes in PBS, the slides were dehydrated in ascending ethanol scales, dried at critical point in CO_2_ (K850 Quorum Technologies, Judges House, Lewes Road, Laughton, East Sussex, BN8 6BN, UK) and then mounted on an aluminum support (stub) with double-sided tape.

Finally, the samples were covered with a gold conductive film in a Cressington 108 sputter coater (Watford, UK) and observed using an SEM Hitachi 400 FEG (Tokyo, Japan).

### 4.7. Analysis of Cell Cycle Phases

The analysis of cell cycle phases was conducted using the NUCLEAR-ID^®^ Red Cell Cycle Kit (ENZ-51008, EuroClone, Via Figino 20/22, Pero (Milan) 20016, Italy) following the manufacturer’s instructions, which are available at https://www.enzolifesciences.com/ENZ-51008/nuclear-id-red-cell-cycle-kit-gfp-certified/, accessed on 18 December 2024). Flow cytometry and ethanol fixed cells were used to conduct this analysis. The ImageStream^®^X Mark II Imaging Flow Cytometer (Merck Life Science Limited, Vale RoadArklowCo, Wicklow, Ireland) was used for the analysis. Moreover, live cell imaging was prepared according to the manufacturer’s instructions, and the image analysis was prepared with Zoe^®^ fluorescence microscopy (Bio-Rad Laboratories, Via Benvenuto Cellini, 18/A, 20054 Segrate (MI), Italy).

#### Dose–Response Analysis

A cell cycle phase analysis was used for the investigation of dose–response curves. The Caco2 cells were cultured for 24 h at 37 °C and CO_2_ (5%) at 1.4 × 10^5^ cells per cell culture flask concentration. After 24 h growth, the cell culture medium was replaced with the cell culture medium containing an increasing concentration of L1. Stock solution of L1 was prepared daily in the complete cell culture medium with 10% fetal bovine serum and sequentially diluted in the complete cell culture medium with 10% fetal bovine serum to obtain a concentration range of 0 (control sample)—0.750 mM. Next, 5 mL of these prepared compound solutions in the cell culture medium were added in triplicate to a cell culture flask where the cells were growing. Successively, the plates were incubated for 24 h. Nocodazole was used as a positive control sample according to the manufacturer’s instructions.

The % Hist of cells arrested in the G2-M phase was calculated as a function of the increasing concentration of L1, which was then used to determine the L1 concentration leading to half of the cell culture population response. The data were analysed with the free calculator available at https://www.aatbio.com/tools/four-parameter-logistic-4pl-curve-regression-online-calculator (accessed on 18 December 2024) using four parameter mode.

### 4.8. Next Generation Sequencing (NGS)

The Caco2 and HEK293T cell samples were cultured using the procedures described in Section 2.3. The negative control samples remained untreated, whereas control samples were treated with 0.13 μM Colcemid solution (final concentration in the cell growing flask) for 24 h. The experimental samples were treated with a 0.74 mM solution (final concentration in the cell growing flask) of L1 for 24 h. Each experiment was performed as a triplicate independent experiment. After 24 h, the cells were harvested and the total RNA extraction was carried out using the automated MagCore Nucleic Acid Extractor system. The purity of the RNA samples was confirmed through spectroscopic analysis using the NanoDrop system.

The NGS and bioinformatic analysis were conducted by the commercial service provided by Centro di Ricerca, Sviluppo e Studi Superiori in Sardegna (http://next.crs4.it/, accessed on 18 December 2024).

### 4.9. Apoptosis/Necrosis Analysis

The analysis of live cells, necrosis, early- and late-apoptosis events was performed using the GFP-CERTIFIED^®^ Apoptosis/Necrosis Detection Kit (ENZ-51002, EuroClone, Via Figino 20/22, Pero (Milan) 20016, Italy) following the manufacturer’s instructions (available at https://www.enzo.com/product/gfp-certified-apoptosis-necrosis-detection-kit/, accessed on 18 December 2024) for flow cytometry with live cells. Nocodazole (final concentration 0.66 μM) was used as a G2/M phase inhibitor in the positive control samples. Each analysis was repeated three times through independent experiments. ImageStream^®^X Mark II Imaging Flow Cytometer was used for the analysis. Moreover, live cell imaging was prepared according to the manufacturer’s instructions, and the image analysis was conducted using Zoe^®^ fluorescence microscopy.

### 4.10. Caspase-3 Activity Assay

The quantitative analysis of Caspese-3 and/or Caspase-7 was prepared with the use of the Caspase-3 activity assay kit (Cell signalling technology, Danvers, MA, USA; product number: #5723) according to the manufacturer’s instructions. In brief, Caco2 and HEK293T cells were seeded in a 96-well plate at 1 × 10^5^ cells/well, and then treated with L1 (24 h treatment, increasing concentrations) and Staurosporine (5 h treatment, increasing concentrations), and then lysate in 30 µL PathSan Sandwich ELISA Lysis Buffer (Cell Signaling Technology, Danvers, MA, USA). Cell lysate was added to assay plates containing substrate solution, and plates were incubated at 37 °C in the dark. Relative fluorescence was acquired at 1 h.

## 5. Conclusions

L1 is a novel molecule that specifically induces mitotic arrest in cancer cells, which is followed by the apoptotic events. Extensive microscopy investigation of cell morphology and quantitative analysis of molecular markers of apoptosis, together with mRNA analysis in NGS studies conducted on both non-tumoural and tumoural cell lines have consistently shown that mitotic arrest occurs exclusively in cancer cells treated with L1. The mechanism of L1 action is selective, which distinguishes L1 from other known antimitotic agents, positioning it as a critical third generation of antimitotic compound. However, the exact mechanism of action is still unknown. The expression analysis of key genes (PNRC1, HERPUD1, TP53INP2, Tob1, and SAT1), which is typically silenced in various tumour tissues and associated with poor prognosis and short survival time, revealed their upregulation upon 24 h treatment with L1. The primary cellular pathways influenced by L1 treatment include the metabolism and biosynthesis of lipids, lipoproteins, steroid, cholesterol, adipogenesis, ferroptosis, ATF-2 and Ap-1 transcription factor network, TGF-beta, IL-18, and VEGFA-VEGFR2. Moreover, L1 treatment downregulates cellular biochemical pathways related to the cell cycle, mitosis, DNA synthesis and repair, and fundamental biochemical cycles (e.g., citric acid cycle, TCA cycle). The mitotic arrest induced by L1 is followed by apoptotic events. Compared to known mitosis inhibitors (Colcemid and Nocodazole), which lack the selectivity, L1 requires higher doses to obtain the mitosis inhibition effect. However, L1 is highly selective towards tumoural cells and does not lead to toxic effects in the non-tumoural cells at the same dose. Given the high water solubility of the L1 molecule and its fast and economic one-step synthesis with respect of green chemistry principles, L1 could start an era of third-generation antimitotic agents that selectively kill tumoural cells, and help to find the processes which distinguish tumoural cells from non-tumoural cells.

## 6. Patents

The invention presented here is patented (patent no. WO202258970_A1).

## Figures and Tables

**Table 1:** Caco2_L1/Caco2_non-treated—10 top DE genes.

External Gene Name	External Gene Source	Description	Base Mean	log2 Fold Change	lfcSE	Stat	*p*-Value	Padj
TUBB2B	HGNC Symbol	tubulin beta 2B class IIb[Source:HGNCSymbol;Acc:HGNC:30829]	4300	1.2	0.05	23	8.70 × 10^−119^	1.20 × 10^−114^
INSIG1	HGNC Symbol	insulin induced gene 1[Source:HGNCSymbol;Acc:HGNC:6083]	2300	1.3	0.063	21	6.10 × 10^−95^	4.30 × 10^−91^
IDI1	HGNC Symbol	isopentenyl-diphosphatedelta isomerase 1[Source:HGNCSymbol;Acc:HGNC:5387]	7700	0.78	0.04	20	1.10 × 10^−86^	5.00 × 10^−83^
PNRC1	HGNC Symbol	proline rich nuclearreceptor coactivator 1[Source:HGNCSymbol;Acc:HGNC:17278]	2700	0.93	0.052	18	8.00 × 10^−71^	2.80 × 10^−67^
HERPUD1	HGNC Symbol	homocysteine inducible ERprotein with ubiquitin likedomain 1 [Source:HGNCSymbol;Acc:HGNC:13744]	4800	0.83	0.047	18	9.80 × 10^−70^	2.80 × 10^−66^
TP53INP2	HGNC Symbol	tumour protein p53inducible nuclear protein 2[Source:HGNCSymbol;Acc:HGNC:16104]	2000	1.2	0.067	17	1.40 × 10^−66^	3.20 × 10^−63^
TOB1	HGNC Symbol	transducer of ERBB2, 1[Source:HGNCSymbol;Acc:HGNC:11979]	8700	0.66	0.039	17	5.10 × 10^−66^	1.00 × 10^−62^
JUN	HGNC Symbol	Jun proto-oncogene, AP-1transcription factorsubunit [Source:HGNCSymbol;Acc:HGNC:6204]	1700	1.2	0.072	17	6.40 × 10^−65^	1.10 × 10^−61^
SAT1	HGNC Symbol	spermidine/spermine N1-acetyltransferase 1[Source:HGNCSymbol;Acc:HGNC:10540]	3800	0.79	0.047	17	1.40 × 10^−64^	2.20 × 10^−61^
TRIB1	HGNC Symbol	tribbles pseudokinase 1[Source:HGNCSymbol;Acc:HGNC:16891]	2000	1.1	0.066	17	2.90 × 10^−62^	4.20 × 10^−59^

**Table 2:** Caco2_Colcemid/Caco2_L1—10 top DE genes.

External Gene Name	External Gene Source	Description	Base Mean	log2 Fold Change	lfcSE	Stat	*p*-Value	Padj
TUBB	HGNC Symbol	tubulin beta class I[Source:HGNCSymbol;Acc:HGNC:20778]	42,000	−2.1	0.072	−29	3.00 × 10^−191^	4.10 × 10^−187^
TUBA1B	HGNC Symbol	tubulin alpha 1b[Source:HGNCSymbol;Acc:HGNC:18809]	73,000	−1.9	0.074	−25	4.40 × 10^−142^	3.00 × 10^−138^
TUBB4B	HGNC Symbol	tubulin beta 4B class IVb[Source:HGNCSymbol;Acc:HGNC:20771]	24,000	−1.8	0.076	−23	9.40 × 10^−122^	4.30 × 10^−118^
ANKRD1	HGNC Symbol	ankyrin repeat domain 1[Source:HGNCSymbol;Acc:HGNC:15819]	6700	2.1	0.1	20	3.70 × 10^−93^	1.30 × 10^−89^
TAGLN	HGNC Symbol	transgelin [Source:HGNCSymbol;Acc:HGNC:11553]	14,000	1.9	0.094	20	1.30 × 10^−91^	3.60 × 10^−88^
ITGA2	HGNC Symbol	integrin subunit alpha 2[Source:HGNCSymbol;Acc:HGNC:6137]	3400	1.9	0.095	20	3.70 × 10^−88^	8.50 × 10^−85^
TUBB2B	HGNC Symbol	tubulin beta 2B class IIb[Source:HGNCSymbol;Acc:HGNC:30829]	4800	−1.4	0.094	−15	1.20 × 10^−51^	2.30 × 10^−48^
LAMA1	HGNC Symbol	laminin subunit alpha 1[Source:HGNCSymbol;Acc:HGNC:6481]	18,000	−1.1	0.074	−15	2.00 × 10^−50^	3.40 × 10^−47^
TUBA1C	HGNC Symbol	tubulin alpha 1c[Source:HGNCSymbol;Acc:HGNC:20768]	27,000	−1.2	0.082	−15	5.60 × 10^−49^	8.50 × 10^−46^
DKK1	HGNC Symbol	dickkopf WNT signalingpathway inhibitor 1[Source:HGNCSymbol;Acc:HGNC:2891]	16,000	1	0.077	13	4.60 × 10^−39^	6.40 × 10^−36^

**Table 3:** HEK293T_Colcemid/HEK293T_non-treated—10 top DE genes.

External_gene_name	External_gene_source	Description	BaseMean	log2FoldChange	lfcSE	Stat	*p*-Value	Padj
TUBB	HGNC Symbol	tubulin beta class I[Source:HGNCSymbol;Acc:HGNC:20778]	56,000	−1.9	0.057	−33	1.4 × 10^−243^	1.7 × 10^−239^
TUBA1B	HGNC Symbol	tubulin alpha 1b[Source:HGNCSymbol;Acc:HGNC:18809]	70,000	−1.7	0.057	−30	3.6 × 10^−195^	2.2 × 10^−191^
FLNA	HGNC Symbol	filamin A [Source:HGNCSymbol;Acc:HGNC:3754]	45,000	1.3	0.057	23	1.3 × 10^−116^	5.1 × 10^−113^
IRS4	HGNC Symbol	insulin receptor substrate4 [Source:HGNCSymbol;Acc:HGNC:6128]	23,000	−1.4	0.063	−22	3.7 × 10^−103^	1.1 × 10^−99^
AL035425.2	Clone-based (Ensembl)gene	insulin receptor substrate4 [Source:NCBIgene;Acc:8471]	9800	−1.7	0.084	−20	4.8 × 10^−93^	1.2 × 10^−89^
AMOT	HGNC Symbol	angiomotin [Source:HGNCSymbol;Acc:HGNC:17810]	51,000	−1.1	0.057	−20	3.4 × 10^−88^	6.8 × 10^−85^
TPM4	HGNC Symbol	tropomyosin 4[Source:HGNCSymbol;Acc:HGNC:12013]	11,000	1.2	0.073	17	1.1 × 10^−63^	1.9 × 10^−60^
GLA	HGNC Symbol	galactosidase alpha[Source:HGNCSymbol;Acc:HGNC:4296]	10,000	−1.3	0.076	−17	2.1 × 10^−62^	3.2 × 10^−59^
TUBB4B	HGNC Symbol	tubulin beta 4B class IVb[Source:HGNCSymbol;Acc:HGNC:20771]	16,000	−1.2	0.072	−16	5.8 × 10^−60^	7.8 × 10^−57^
JUN	HGNC Symbol	Jun proto-oncogene, AP-1transcription factorsubunit [Source:HGNCSymbol;Acc:HGNC:6204]	9500	1.2	0.076	15	8.8 × 10^−53^	1.1 × 10^−49^

**Table 4:** HEK293T_L1/HEK293T_non-treated—10 top DE genes.

External_gene_name	External_gene_source	Description	BaseMean	log2FoldChange	lfcSE	Stat	*p*-Value	Padj
IRS4	HGNC Symbol	insulin receptor substrate4 [Source:HGNCSymbol;Acc:HGNC:6128]	23,000	−0.69	0.032	−22	8.8 × 10^−106^	1.2 × 10^−101^
NEFM	HGNC Symbol	neurofilament medium[Source:HGNCSymbol;Acc:HGNC:7734]	15,000	−0.61	0.035	−18	2.4 × 10^−70^	1.7 × 10^−66^
BMP2	HGNC Symbol	bone morphogeneticprotein 2 [Source:HGNCSymbol;Acc:HGNC:1069]	4100	−0.88	0.051	−17	6.8 × 10^−66^	3.1 × 10^−62^
AL035425.2	Clone-based (Ensembl)gene	insulin receptor substrate4 [Source:NCBIgene;Acc:8471]	9400	−0.86	0.055	−16	6.3 × 10^−55^	2.2 × 10^−51^
ID2	HGNC Symbol	inhibitor of DNA binding2 [Source:HGNCSymbol;Acc:HGNC:5361]	7200	−0.74	0.048	−16	2.9 × 10^−54^	8 × 10^−51^
FAM84B	HGNC Symbol	family with sequencesimilarity 84 member B[Source:HGNCSymbol;Acc:HGNC:24166]	4500	−0.86	0.059	−15	1 × 10^−48^	2.3 × 10^−45^
DFFA	HGNC Symbol	DNA fragmentation factorsubunit alpha[Source:HGNCSymbol;Acc:HGNC:2772]	12,000	−0.54	0.037	−14	2.2 × 10^−47^	4.3 × 10^−44^
TFRC	HGNC Symbol	transferrin receptor[Source:HGNCSymbol;Acc:HGNC:11763]	15,000	0.5	0.037	14	8.7 × 10^−42^	1.5 × 10^−38^
AMOT	HGNC Symbol	angiomotin[Source:HGNCSymbol;Acc:HGNC:17810]	56,000	−0.34	0.026	−13	1 × 10^−40^	1.6 × 10^−37^
CA2	HGNC Symbol	carbonic anhydrase 2[Source:HGNCSymbol;Acc:HGNC:1373]	15,000	−0.5	0.038	−13	6 × 10^−38^	8.3 × 10^−35^

## Data Availability

The datasets used and/or analyzed during the current study are available from the corresponding author upon reasonable request.

## Supplementary Materials

The following supporting information can be downloaded at: https://www.mdpi.com/article/10.3390/ph18010011/s1, Physico-chemical characterisation of L1, L2, and L3 synthesis products; Figure S1. Flow cytometry dot plots of Caco-2 cells after 24 h of growth: (A) without any treatment, (B) with L1, and (C) with Staurosporine. Caco-2 cells after 48 h of growth: (D) without any treatment, (E) with L1, and (F) with Staurosporine; Figure S2. Red cell cycle visualisation in Caco2 cells treated with L1 (0.74mM) or Staurosporine (0.400 µM). Bar = 100 µm; Figure S3. Caspase-3 quantitative analysis of HEK293T cell lysate after 24 h treatment with growing concentrations of L1 (A) and 5 h treatment with growing concentrations of Staurosporine (B). The data are presented as the mean ± SD (*n* = 3). Significant statistical differences between control samples (concentration = 0 [µM]) and experimental samples were identified as *p* < 0.05; Figure S4. Cytoflow analysis of cell cycle phase distribution in Caco-2 (A-C) and 293T (D-F) cells. Representative flow cytometry charts of (A–C) Caco-2 cells; (D–F) 293T cells; showing untreated cells, cells treated with Nocodazole (positive control), and cells treated with L1 (0.74 mM) for 24 h, respectively. The cytoflow analysis was performed using the NUCLEAR-ID^®^ Red Cell Cycle Kit of Enzo, following the manufacturer instructions; Table S1. EnrichmentAnalysis_toppCluster_DE_gene upregulated by the L1 24-h treatment in Caco2; Table S2. EnrichmentAnalysis_toppCluster_DE_gene downregulated by the L1 24 h treatment in Caco2 cells; Figure S5. LunaFL analysis of total cell number, viability, and cell size using Trypan Blue staining in Caco2 cells (A–C) and U118 (D–F); Figure S6. The graphs depict (A) the time course of cell number in Caco2 cells treated with L1 (grey; L1 concentration: 0.74 mM).) compared to the corresponding control (black; untreated cells) as measured by the LunaFL automatic cell counting system. (B) The time course of viability percentages in Caco2 cells treated with L1 (grey; L1 concentration: 0.74 mM) compared to the corresponding control (black; untreated cells) using the LunaFL automatic cell counting system. Data are presented as the mean ± SD (*n* = 3). αP < 0.05, βP < 0.01 indicate significant differences in cell number compared to the respective values in cells not exposed to L1; Figure S7. Cytoflow analysis of cell cycle phase distribution (representative data of three independent replicates of experiment) in Caco-2 and increasing L1 concentration in cell culture medium (A) 0 mM; (B) 0.7500 mM; (C) 0.3750; (D) 0.1875 mM; (E) 0.0750 mM; (F) 0.0375; (G) 0.0150; (H) 0.0075; (I) 0.0015 mM; (J) 0.0007 mM; Table S3. Mitotic index calculation.
