# Supplementary material for: Kojic Acid Derivative as an Antimitotic Agent That Selectively Kills Tumour Cells
Source: Pharmaceuticals (Basel). 2024 Dec 25;18(1):11. doi: 10.3390/ph18010011 (PMC11768441; doi:10.3390/ph18010011)
Supplement: Supplementary file 1 [file pharmaceuticals-18-00011-s001.zip › pharmaceuticals-3267163-supplementary.pdf]

Kojic acid derivative as an antimitotic agent that selectively kills tumour cells

Giuseppina Pichiri,[1] Marco Piludu,[2] Terenzio Congiu,[1] Nicole Grandi,[3] Pierpaolo Coni,[1] Monica Piras,[1] Mariusz Jaremko,[4] Joanna Izabela Lachowicz,[1,5*]

[1] Department of Medical Sciences and Public Health, University of Cagliari, Cittadella Universitaria, 09042 Monserrato (CA), Italy; pichiri@unica.it; terenzio.congiu@unica.it; monica.piras@unica.it; lachowicz@unica.it

[2] Department of Biomedical Sciences, University of Cagliari, Cittadella Universitaria, 09042 Monserrato (CA), Italy; mpiludu@unica.it

[3] Department of Life and Environmental Sciences, University of Cagliari, Cittadella Universitaria, 09042 Monserrato (CA), Italy; Nicole.grandi@unica.it

[4] Smart-Health Initiative (SHI) and Red Sea Research Center (RSRC), Division of Biological and Environmental Sciences and Engineering (BESE), King Abdullah University of Science and Technology (KAUST), Thuwal 23955-6900, Saudi Arabia; Mariusz.jaremko@kaust.edu.sa

[5] Department of Population Health, Division of Environmental Health, Occupational Medicine and Epidemiology, Wroclaw Medical University, Mikulicza-Radeckiego 7, Wroclaw, PL 50-368, Poland; joanna.lachowicz@umw.edu.pl

* Correspondence: lachowicz@unica.it;

Sommario

Physico-chemical characterization of L1, L2, and L3 synthesis products 3

2,2'-[ethane-1,2-diylbis(iminomethanediyl)]bis(5-hydroxy-4H-pyran-4-one) (L1) 3

2,2'-[propane-1,3-diylbis(iminomethanediyl)]bis(5-hydroxy-4H-pyran-4-one) (L2) 3

2,2'-[butane-1,4-diylbis(iminomethanediyl)]bis(5-hydroxy-4H-pyran-4-one) (L3) 3

6,6',6''-(((nitrilotris(ethane-2,1-diyl))tris(azanediyl))tris(methylene))tris(3-hydroxy-4H-pyran-4-one) (L4) 3

Figure S1. Flow cytometry dot plots of Caco-2 cells after 24 hours of growth: **(A)** without any treatment, **(B)** with L1, and **(C)** with Staurosporine**.** Caco-2 cells after 48 hours of growth: **(D)** without any treatment, **(E)** with L1, and **(F)** with Staurosporine**.** 4

Figure S2. Red cell cycle visualization in Caco2 cells treated with L1 (0.74mM) or Staurosporine (0.400 µM). Bar = 100 um. 5

Figure S3. Caspase-3 quantitative analysis of HEK293T cell lysate after 24-hours treatment with growing concentrations of L1 (A) and 5-hours treatment with growing concentrations of Staurosporine (B). The data are presented as the mean ± SD (n = 3). Significant statistical differences between control samples (concentration = 0 [µM]) and experimental samples were identified as $p < 0.05$. 6

Figure S4. Cytoflow analysis of cell cycle phase distribution in Caco-2 (A-C) and 293T (D-F) cells. Representative flow cytometry charts of A-C) Caco-2 cells; D-F) 293T cells; showing untreated cells, cells treated with Nocodazole (positive control), and cells treated with L1 (0.74 mM) for 24 hours, respectively. The cytoflow analysis was performed using the NUCLEAR-ID® Red Cell Cycle Kit of Enzo, following the manufacturer instructions. 7

Table S1. EnrichmentAnalysis_toppCluster_DE_gene upregulated by the L1 24-hour treatment in Caco2 cells 8

Table S2. EnrichmentAnalysis_toppCluster_DE_gene downregulated by the L1 24-hour treatment in Caco2 cells 13

Figure S5. Luna^{FL} analysis of total cell number, viability, and cell size using Trypan Blue staining in Caco2 cells (A-C) and U118 (D-F). 24

Figure S6. The graphs depict **(A)** the time course of cell number in Caco2 cells treated with L1 (grey colour; L1 concentration: 0.74 mM).) compared to the corresponding control (black colour; untreated cells) as measured by the Luna^{FL} automatic cell counting system. **(B)** The time course of viability percentages in Caco2 cells treated with L1 (grey colour; L1 concentration: 0.74 mM) compared to the corresponding control (black colour; untreated cells) using the Luna^{FL} automatic cell counting system. Data are presented as the mean ± SD (n = 3). $^{\alpha}P < 0.05$, $^{\beta}P < 0.01$; indicating significant differences in cell number compared to the respective values in cells not exposed to L1. 25

Figure S7. Cytoflow analysis of cell cycle phase distribution (representative data of 3 independent replicates of experiment) in Caco-2 and increasing L1 concentration in cell culture medium (A) 0 mM; (B) 0.7500 mM; (C) 0.3750; (D) 0.1875 mM; (E) 0.0750 mM; (F) 0.0375; (G) 0.0150; (H) 0.0075; (I) 0.0015 mM; (J) 0.0007 mM 26

Table S3. Mitotic index calculation. 27

Physico-chemical characterization of L1, L2, and L3 synthesis products

2,2'-[ethane-1,2-diylbis(iminomethanediyl)]bis(5-hydroxy-4H-pyran-4-one) (L1)

1.0 g (7 mmol) of KA was dissolved in 20 mL of ethanol (96%), followed by the addition of 230 µl (3.5 mmol) of ethylenediamine. The reaction mixture was stirred at room temperature for 1 hour, and the resulting precipitate was filtered and washed with ethyl acetate. The identity and purity of the sample were confirmed using NMR analysis. Ligand S2 – Yield 98%, Melting Point 138°C, ^{1}H NMR (500 Hz, D_2O), δ (ppm): 7.80 (s, 1H, 6-HPy), 6.42 (s, 1H, 3-HPy), 4.39 (s, 2H, CH_2–Py), 2.95 (s, 2H, CH_2).

2,2'-[propane-1,3-diylbis(iminomethanediyl)]bis(5-hydroxy-4H-pyran-4-one) (L2)

1.0 g (7 mmol) of KA was dissolved in 20 mL of ethanol (96%), and 290 µl (3.5 mmol) of propane-1,3-diamine was added. The reaction mixture was stirred at room temperature for 1 hour, and the resulting precipitate was filtered and washed with ethyl acetate. The identity and purity of the sample were confirmed by NMR analysis. Ligand S4 – Yield 91%, Melting Point 145°C, ^{1}H NMR (500 Hz, D_2O), δ (ppm): 7.71 (s, 1H, 6-HPy), 6.38 (s, 1H, 3-HPy), 4.38 (s, 2H, CH_2–Py), 2.95-2.94-2.92 (t, 2H, CH_2), 1.91-1.90-1.88-1.87-1.85 (m, 2H, CH_2).

2,2'-[butane-1,4-diylbis(iminomethanediyl)]bis(5-hydroxy-4H-pyran-4-one) (L3)

1.0 g (7 mmol) of KA was dissolved in 20 mL of ethanol (96%), and 250 µl (3.5 mmol) of butane-1,4-diamine was added. The reaction mixture was stirred at room temperature for 1 hour, and the resulting precipitate was filtered and washed with ethyl acetate. The identity and purity of the sample were confirmed by NMR analysis. Ligand S5 – Yield 94%, Melting Point 168°C, ^{1}H NMR (500 Hz, D_2O), δ (ppm): 7.68 (s, 1H, 6-HPy), 6.36 (s, 1H, 3-HPy), 4.37 (s, 2H, CH_2–Py), 2.91 (s, 2H, CH_2) , 1.63 (s, 2H, CH_2).

6,6',6''-(((nitrilotris(ethane-2,1-diyl))tris(azanediyl))tris(methylene))tris(3-hydroxy-4H-pyran-4-one) (L4)

Kojic acid (1.0 g, 7.04 mmol) was suspended in 20 mL ethanol followed by the dropwise addition of tris(2-aminoethyl)amina (350 µL, 2.32 mmol) dissolved in $CHCl_3$ (2 mL). The reaction mixture was stirred at room temperature for 1 hour, and the resulting precipitate was filtered and washed with ethyl acetate. The identity and purity of the sample were confirmed by NMR and elemental analysis. Analytical data: Yield 98%, 1H NMR (D2O, 500 MHz) δ 2.83 (t, 6H, J = 2.81 Hz), 3.09 (t, 6H, J = 3.09 Hz), 4.49 (s, 6H), 6.49 (s, 3H), 7.82 (s, 3H). 13C NMR (D2O, 500 MHz) δ 39.84, 53.86, 62.92, 112.94, 145.16, 168.24, 184.01, 199.25. Elemental analysis (%) calculated for (C24H36N4O12·3H2O): C 50.35; H 6.34, N 9.79 %; found: C 50.65, H 6.17, N 9.27 %.

Figure S1. Flow cytometry dot plots of Caco-2 cells after 24 hours of growth: **(A)** without any treatment, **(B)** with L1, and **(C)** with Staurosporine**.** Caco-2 cells after 48 hours of growth: **(D)** without any treatment, **(E)** with L1, and **(F)** with Staurosporine**.**

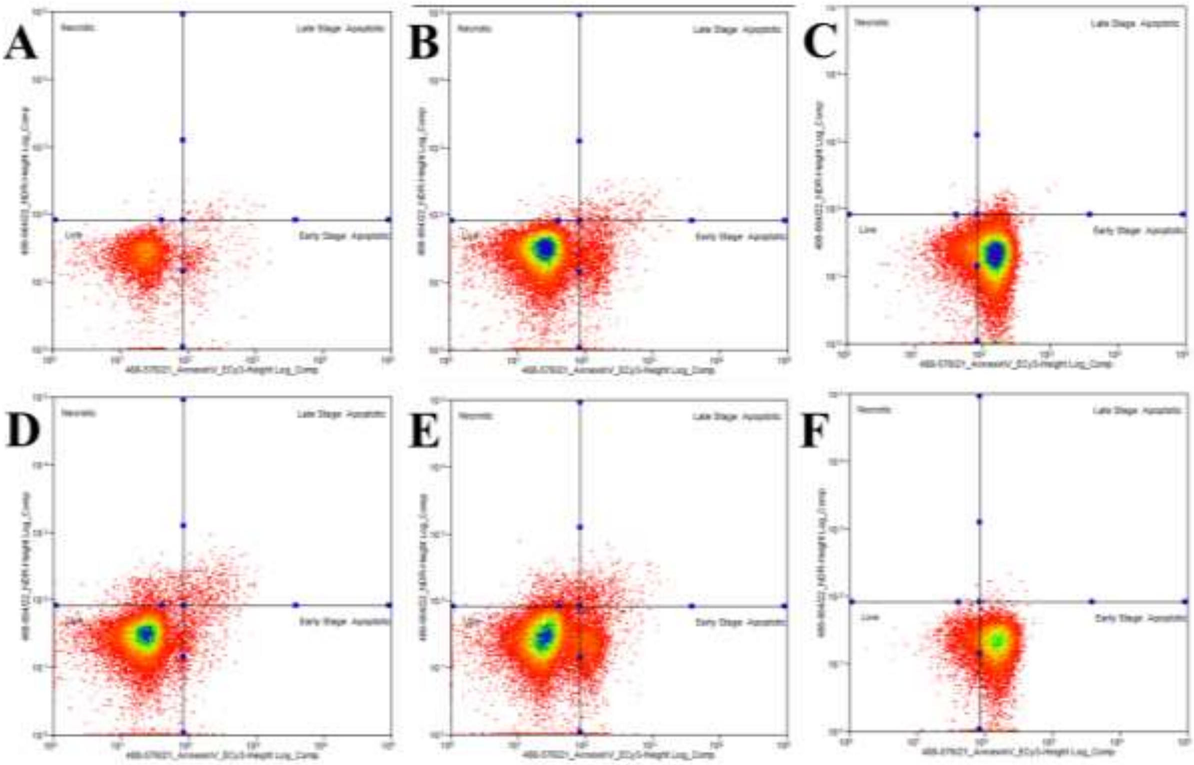

Figure S2. Red cell cycle visualization in Caco2 cells treated with L1 (0.74mM) or Staurosporine (0.400 µM). Bar = 100 um.

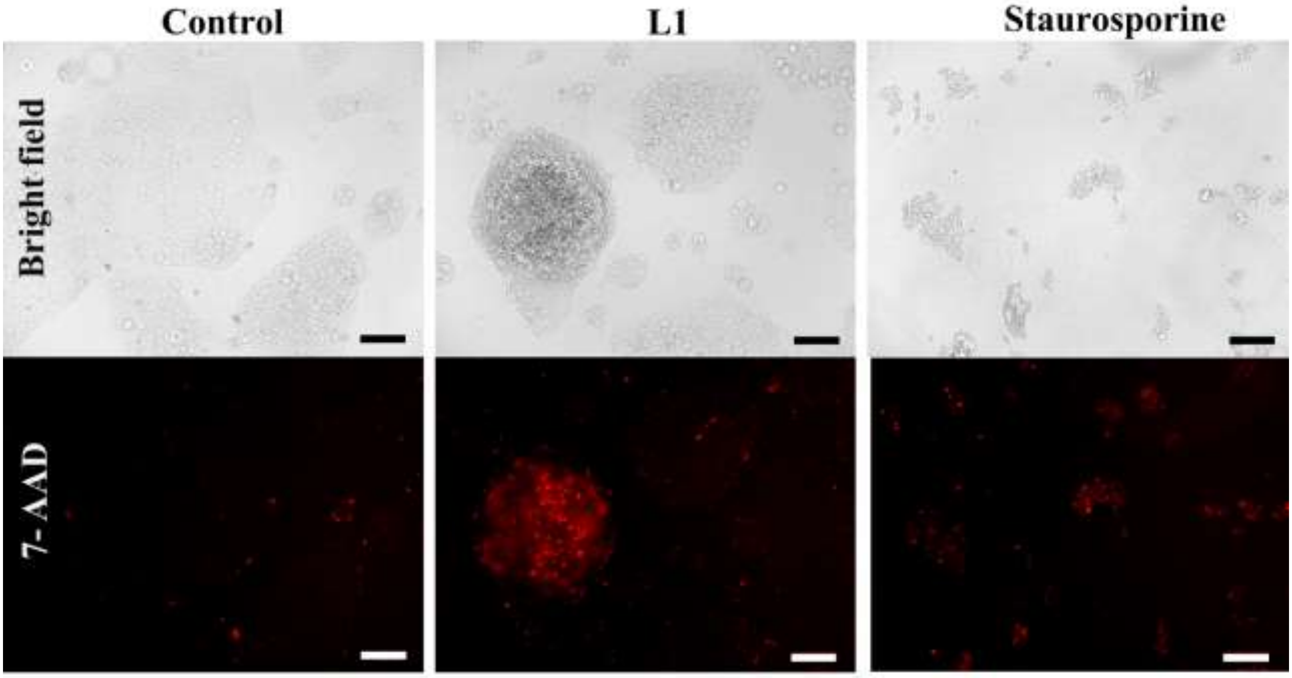

Figure S3. Caspase-3 quantitative analysis of HEK293T cell lysate after 24-hours treatment with growing concentrations of L1 (A) and 5-hours treatment with growing concentrations of Staurosporine (B). The data are presented as the mean ± SD (n = 3). Significant statistical differences between control samples (concentration = 0 [µM]) and experimental samples were identified as $p < 0.05$.

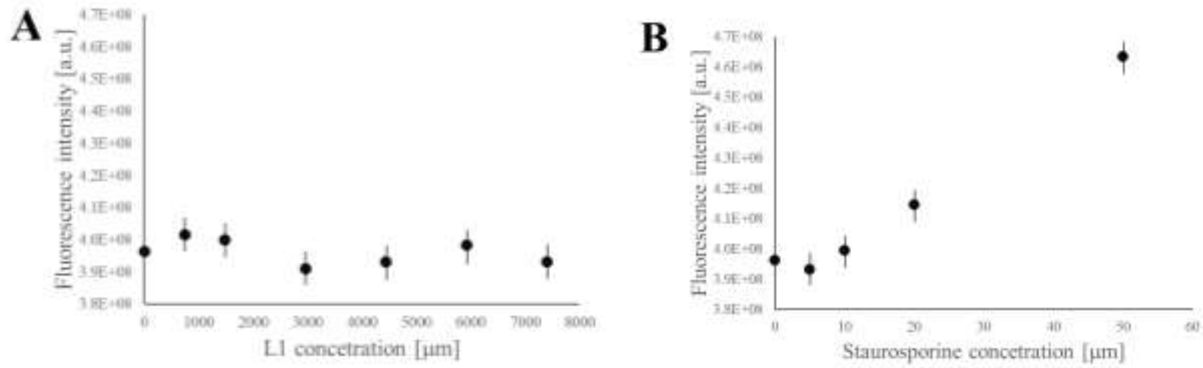

Figure S4. Cytoflow analysis of cell cycle phase distribution in Caco-2 (A-C) and 293T (D-F) cells. Representative flow cytometry charts of A-C) Caco-2 cells; D-F) 293T cells; showing untreated cells, cells treated with Nocodazole (positive control), and cells treated with L1 (0.74 mM) for 24 hours, respectively. The cytoflow analysis was performed using the NUCLEAR-ID® Red Cell Cycle Kit of Enzo, following the manufacturer instructions.

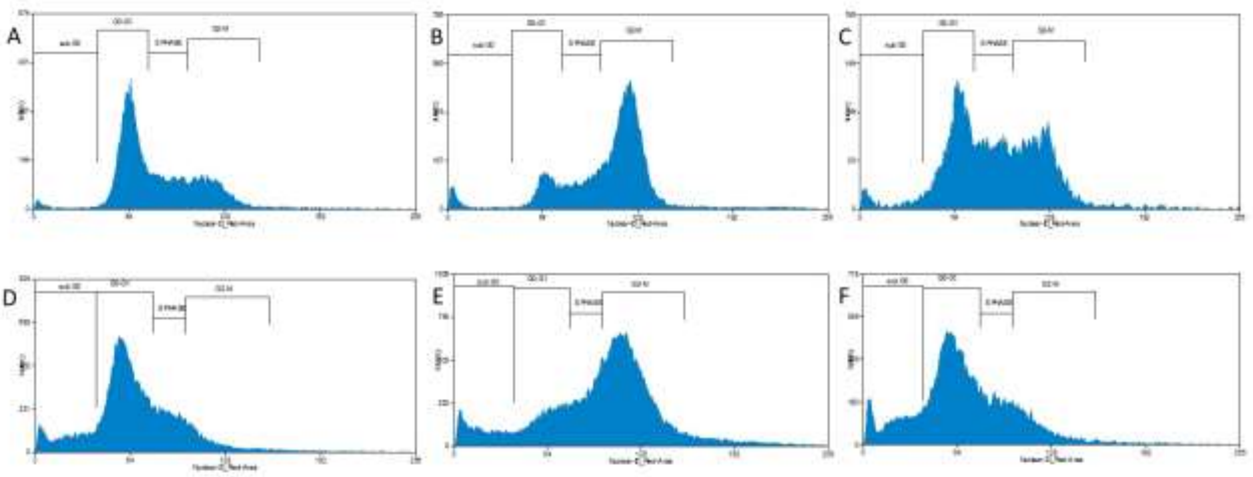

Table S1. EnrichmentAnalysis_toppCluster_DE_gene upregulated by the L1 24-hour treatment in Caco2 cells

Verbose ID	Caco2_L1_vs_Caco2_non-treated_UP_in_Caco2_S2_GeneSet; -LOG10 = 10
**Pathway_1269650_ Generic Transcription Pathway**	AGO4,ARID3A,ATF2,BBC3,BCL2L14,BCL6,BIRC5,BNIP3L,BRPF3,BTG2,CCNK,CDK7,CDK9,CDKN1A,CDKN2B,CITED2,CITED4,CNOT4,CSNK2A1,CSNK2A2,DDIT4,EP300,ERBB2,FOS,GADD45A,GLS2,GPX2,GTF2H5,HNF4A,JMY,JUN,JUNB,KCTD15,KDM5B,MAPK11,MDC1,MED1,MED13,MED17,MED20,NCOA6,NCOR2,NEDD4L,NELFCD,NOTCH1,NR1D1,NR1D2,NR1I3,NR2C1,NR2C2,NR2E1,NR2F1,NR2F6,NR4A1,NR4A2,NR4A3,NR5A2,NRBF2,NRBP1,NUAK1,PDPK1,PLK2,PMAIP1,POLR2A,POLR2G,PPARG,PPM1A,PPP1R13B,PTEN,RGCC,RHEB,RNF111,RRAGD,RXRB,SESN1,SESN2,SESN3,SGK1,SKIL,SMAD7,SUPT4H1,SUPT5H,TAF7,TAF9,TEAD2,TEAD3,TFAP2C,TGIF1,THRA,TNFRSF10B,TNKS1BP1,TNRC6A,TNRC6B,TP53,TP53BP2,TP53I3,TP53INP1,TRIM28,UBB,VEGFA,WRN,WWTR1,YAP1,YWHAH,YWHAZ,ZFP1,ZFP14,ZKSCAN5,ZKSCAN8,ZNF10,ZNF12,ZNF140,ZNF155,ZNF160,ZNF184,ZNF205,ZNF211,ZNF226,ZNF227,ZNF23,ZNF230,ZNF254,ZNF266,ZNF267,ZNF273,ZNF274,ZNF282,ZNF285,ZNF3,ZNF317,ZNF33A,ZNF33B,ZNF350,ZNF354A,ZNF383,ZNF385A,ZNF419,ZNF426,ZNF432,ZNF436,ZNF446,ZNF461,ZNF468,ZNF480,ZNF486,ZNF496,ZNF510,ZNF529,ZNF544,ZNF551,ZNF562,ZNF563,ZNF566,ZNF595,ZNF600,ZNF607,ZNF614,ZNF615,ZNF616,ZNF619,ZNF664,ZNF689,ZNF697,ZNF703,ZNF706,ZNF707,ZNF710,ZNF711,ZNF721,ZNF74,ZNF761,ZNF771,ZNF773,ZNF777,ZNF778
**Pathway_1270001_ Metabolism of lipids and lipoproteins**	AACS,ABHD4,ACACB,ACAT2,ACBD5,ACHE,ACOT1,ACOT13,ACOT9,ACP6,ACSF3,ACSL3,ACSL4,ACSL5,AGPAT2,AGPAT3,AGPAT4,AKR1C1,APOA1,APOC1,ARNTL,ARSB,ARSL,ASAH2,B4GALNT1,BAAT,CAV1,CHKB,CIDEC,CPT1A,CREB3L3,CSNK2A1,CSNK2A2,CYP17A1,CYP1A1,CYP27A1,CYP51A1,DBI,DEGS1,DHCR7,EBP,ELOVL3,ELOVL4,ELOVL5,EP300,FDFT1,FDPS,FHL2,FITM2,FURIN,FYN,GDE1,GDPD5,GGPS1,GPX2,GPX4,GRHL1,HMGCR,HMGCS1,HSD17B7,IDH1,IDI1,INPP5J,INSIG1,LDLR,LDLRAP1,LIPA,LIPE,LIPH,LPCAT2,LPIN1,LPIN2,LSS,MAPKAPK2,MED1,MED13,MED13L,MED17,MED20,MED21,MGLL,MID1IP1,MSMO1,MTMR14,MTMR3,MVD,MVK,MYLIP,NCOA6,NCOR2,NEU1,NFYB,NPC1,NR1D1,NSDHL,ORMDL3,OSBPL10,OSBPL2,OSBPL3,OSBPL6,PCSK9,PCYT1B,PHOSPHO1,PI4K2A,PIK3C2B,PIK3R1,PLAAT3,PLEKHA3,PLEKHA4,PLIN2,PLIN3,PLPP1,PLPP3,PNPLA2,PNPLA3,PNPLA6,PPARG,PPM1L,PPT2,PRKD2,PTEN,PTGES2,PTGR1,PTGS2,RAB5A,SC5D,SCD,SCD5,SGMS1,SH3KBP1,SOAT2,SQLE,SRD5A3,SREBF2,SULT2A1,SYNJ2,TEAD2,TEAD3,TIAM2,TNFAIP8,TRIB3,WWTR1,YAP1,ZDHHC8
**Pathway_1270037_ Cholesterol biosynthesis**	ACAT2,CYP51A1,DHCR7,EBP,FDFT1,FDPS,GGPS1,HMGCR,HMGCS1,HSD17B7,IDI1,LSS,MSMO1,MVD,MVK,NSDHL,SC5D,SQLE
**Pathway_1270038_ Regulation of cholesterol biosynthesis by SREBP (SREBF)**	ACACB,CYP51A1,DHCR7,FDFT1,FDPS,GGPS1,HMGCR,HMGCS1,IDI1,INSIG1,LSS,MED1,MVD,MVK,NCOA6,NFYB,SC5D,SCD,SQLE,SREBF2
**Pathway_1270039_ Activation of gene expression by SREBF (SREBP)**	ACACB,CYP51A1,DHCR7,FDFT1,FDPS,GGPS1,HMGCR,HMGCS1,IDI1,LSS,MED1,MVD,MVK,NCOA6,NFYB,SC5D,SCD,SQLE,SREBF2

**Pathway_137939_Direct p53 effectors**	ARID3A,ATF3,BBC3,BCL2L14,BCL6,BNIP3L,BTG2,CAV1,CCNK,CDKN1A,DDIT4,DUSP1,DUSP5,EP300,GADD45A,GDF15,IRF5,JMY,JUN,LIF,MAP4K4,NFYB,PMAIP1,PPP1R13B,PTEN,RGCC,SESN1,TAF9,TAP1,TNFRSF10B,TP53,TP53BP2,TP53I3,TP53INP1,ZNF385A
**Pathway_138006_ATF-2 transcription factor network**	ACHE,ATF2,ATF3,CSRP2,CXCL8,DUSP1,DUSP10,DUSP5,DUSP8,EP300,FOS,GADD45A,JDP2,JUN,JUNB,JUND,MAPK11,MAPK8,NOS2,PDGFRA,PLAU,SOCS3
**Pathway_142266_cholesterol biosynthesis II (via 24,25-dihydrolanosterol)**	CYP51A1,DHCR7,EBP,FDFT1,HSD17B7,LSS,MSMO1,NSDHL,SC5D,SQLE
**Pathway_142267_cholesterol biosynthesis I**	CYP51A1,DHCR7,EBP,FDFT1,HSD17B7,LSS,MSMO1,NSDHL,SC5D,SQLE
**Pathway_142268_cholesterol biosynthesis III (via desmosterol)**	CYP51A1,DHCR7,EBP,FDFT1,HSD17B7,LSS,MSMO1,NSDHL,SC5D,SQLE
**Pathway_142269_superpathway of cholesterol biosynthesis**	ACAT2,CYP51A1,DHCR7,EBP,FDFT1,FDPS,GGPS1,HMGCR,HMGCS1,HSD17B7,IDI1,LSS,MSMO1,MVD,MVK,NSDHL,SC5D,SQLE
**Pathway_1496831_Mitophagy - animal**	ATF4,ATG9A,ATG9B,BNIP3,BNIP3L,CITED2,CSNK2A1,CSNK2A2,FOXO3,GABARAPL1,JUN,MAPK8,OPTN,PINK1,RELA,RRAS,SQSTM1,TAX1BP1,TBC1D17,TFE3,TFEB,TP53,UBB,ULK1
**Pathway_1510435_Ferroptosis**	ACSL3,ACSL4,ACSL5,GCLC,GCLM,GPX4,HMOX1,MAP1LC3B,PCBP1,PCBP2,PRNP,SAT1,SLC11A2,SLC3A2,SLC40A1,SLC7A11,TP53
**Pathway_413390_Cholesterol biosynthesis, squalene 2,3-epoxide => cholesterol**	CYP51A1,DHCR7,EBP,FAXDC2,HSD17B7,LSS,MSMO1,NSDHL,SC5D
**Pathway_82937_Steroid biosynthesis**	CYP51A1,DHCR7,EBP,FAXDC2,FDFT1,HSD17B7,LIPA,LSS,MSMO1,NSDHL,SC5D,SOAT2,SQLE

**Pathway_M166_ATF-2 transcription factor network**	ACHE,ATF2,ATF3,CSRP2,CXCL8,DUSP1,DUSP10,DUSP5,DUSP8,EP300,FOS,GADD45A,JDP2,JUN,JUNB,JUND,MAPK11,MAPK8,NOS2,PDGFRA,PLAU,SOCS3
**Pathway_M167_AP-1 transcription factor network**	ATF2,ATF3,BCL2L11,CXCL8,DUSP1,EDN1,EGR1,ELF1,EP300,FOS,FOSB,FOSL2,GATA2,JUN,JUNB,JUND,MAF,MAFG,NFATC2,PLAU,PTEN,TCF7L2,TGFB1,TP53
**Pathway_M229_Signaling mediated by p38-alpha and p38-beta**	ATF2,ATF6,CEBPB,CSNK2A1,CSNK2A2,EIF4EBP1,HBP1,JUN,KRT19,MAPK11,MAPKAPK2,MEF2A,NOS2,PTGS2,RAB5A,SLC9A1,TP53
**Pathway_M39432_TGF-beta Signaling Pathway**	ATF2,ATF3,AXIN1,CAV1,CCNB2,CDKN1A,CDKN2B,CUL1,DAB2,EP300,FOS,FOSB,JUN,JUNB,JUND,KLF10,KLF11,KLF6,MAPK8,MEF2A,NEDD4L,NEDD9,PIK3R1,PJA1,PPM1A,PTK2,RNF111,SKIL,SMAD7,SMURF1,SOS1,TGFB1,TGFB1I1,TGIF1,TP53,YAP1
**Pathway_M39505_Adipogenesis**	AGPAT2,BMP2,CDKN1A,CEBPA,CEBPB,CEBPD,CYP26A1,EGR2,EPAS1,GADD45A,GADD45B,GATA2,GATA3,IRS1,IRS2,KLF5,KLF6,LIF,LIPE,LPIN1,LPIN2,MEF2A,MEF2D,MIF,NCOR2,NR2F1,PCK2,PLIN2,PNPLA3,PPARG,SCD,SOCS3,TGFB1,TRIB3,WWTR1
**Pathway_M39608_Cholesterol Biosynthesis Pathway**	CYP51A1,DHCR7,FDFT1,FDPS,HMGCR,HMGCS1,IDI1,LSS,MSMO1,MVD,MVK,NSDHL,SC5D,SQLE
**Pathway_M39729_VEGFA-VEGFR2 Signaling Pathway**	ACACB,ACOT9,ADAMTS9,ANXA1,ATF2,ATF4,ATF6,BIRC5,BMP2,C15orf39,CAPN2,CAV1,CFL1,CHAC1,CRIP2,CSRP1,CSRP2,CXCL8,DLL4,DNAJA1,DNAJB9,DUSP5,EGR1,EGR3,EZR,FGB,FGG,FHL2,FOXO3,FOXO4,FSCN1,FUT1,FYN,GAB1,GATA2,GIPC1,GRB10,GSK3B,HBEGF,HDAC5,HDAC7,HERPUD1,IER5,JUN,LRRFIP2,MAPK8,MAPKAPK2,MMP14,NCK1,NFATC2,NFKBIA,NR4A1,NR4A2,NR4A3,NRARP,NUMB,OCLN,P4HA2,PABPC1,PAK1,PBXIP1,PDPK1,PGD,PGF,PIK3R1,PLAU,PLCG1,PRKCE,PRKD2,PTGS2,PTK2,PTK2B,RAB5A,RBM39,RCN1,RELA,RND1,SDCBP,SH3BGRL3,SHB,SLC2A14,SMARCA2,SPIRE1,SRF,TMEM170A,TMSB10,TMSB4X,TNFRSF25,TNXB,TRIP4,VEGFA
**Pathway_M39768_Ferroptosis**	ACSL3,ACSL4,ACSL5,GCLC,GCLM,GPX4,HMOX1,MAP1LC3B,PCBP1,PCBP2,PRNP,SAT1,SLC11A2,SLC3A2,SLC40A1,SLC7A11,TP53
**Pathway_M39818_IL-18 signaling pathway**	AARS1,ACACB,ATF3,BIRC3,BMP2,BTG2,CA11,CCL20,CCNB2,CD81,CD83,CEBPB,CFLAR,CLDN3,CLDN4,COL1A1,COX17,CPT1A,CXCL16,CXCL8,EPS8,FOS,FUT1,GSK3B,HMOX1,HOXD8,HSPB8,IL18,IRF1,IRF6,JUN,KCNH2,MEF2A,MEPCE,MMP14,NFKB2,NFKBIA,NOS2,NPPB,NR4A1,PIK3R1,PKN1,PLCG1,PPT2,PRCC,PTEN,PTGS2,PTMS,RELA,RND2,RXRB,SDC4,SEMA6C,SOCS3,TMSB4X,TP53,ULBP2,VEGFA,ZC3H12A,ZNF219
**Pathway_M39853_Cholesterol metabolism (includes both**	ACSL3,ACSL4,CYP27A1,CYP51A1,DHCR7,EBP,ELOVL3,ELOVL4,ELOVL5,FDFT1,FDPS,GGPS1,HMGCR,HMGCS1,HSD17B7,IDI1,LSS,MSMO1,MVD,MVK,MYLIP,NSDHL,SC5D,SCD,SOAT2,SQLE,SREBF2

**Bloch and Kandutsch-Russell pathways)**	
**Pathway_M39866_ Gastrin Signaling Pathway**	ATF2,BIRC3,BIRC5,BMP2,CDKN1A,CXCL8,EGR1,EIF4EBP1,FOS,FOXO3,FYN,GSK3B,HDAC7,IRS1,JUN,KLF4,MAP3K11,MAPK8,MEF2D,NFKBIA,PAK1,PIK3R1,PLCG1,PPARG,PRKCE,PRKCQ,PRKD2,PTGS2,PTK2,RELA,RHOB,SLC9A1,SOS1,VEGFA
**Pathway_M5872_Steroid biosynthesis**	CYP51A1,DHCR7,EBP,FDFT1,HSD17B7,LIPA,LSS,MSMO1,NSDHL,SC5D,SOAT2,SQLE
**Pathway_MAP00100_Sterol_biosynthesis_MAP00100 Sterol biosynthesis**	FDFT1,FDPS,HMGCR,IDI1,LSS,MVD,MVK,SC5D,SQLE
**Pathway_PW:0000248_steroid biosynthetic**	DHCR7,FDFT1,FDPS,IDI1,LSS,MVD,MVK,SQLE
**Pathway_PW:0000454_cholesterol biosynthetic**	ACAT2,CYP51A1,DHCR7,EBP,FDFT1,FDPS,HMGCR,HMGCS1,IDI1,LSS,MVD,MVK,NSDHL,SC5D,SQLE
**Pathway_SMP00023_Steroid Biosynthesis**	ACAT2,CYP51A1,EBP,FDFT1,FDPS,GGPS1,HMGCR,HMGCS1,HSD17B7,IDI1,LIPA,LSS,MSMO1,MVD,MVK,NSDHL,SQLE
**Pathway_SMP00079_Ibandronate Pathway**	ACAT2,FDFT1,FDPS,GGPS1,HMGCR,HMGCS1,LSS,MVD,MVK,SQLE
**Pathway_SMP00089_Pravastatin Pathway**	ACAT2,FDFT1,FDPS,GGPS1,HMGCR,HMGCS1,LSS,MVD,MVK,SQLE
**Pathway_SMP00092_Rosuvastatin Pathway**	ACAT2,FDFT1,FDPS,GGPS1,HMGCR,HMGCS1,LSS,MVD,MVK,SQLE
**Pathway_SMP00095_Alendronate pathway**	ACAT2,FDFT1,FDPS,GGPS1,HMGCR,HMGCS1,LSS,MVD,MVK,SQLE

**Pathway_SMP00099_Lovastatin Pathway**	ACAT2,FDFT1,FDPS,GGPS1,HMGCR,HMGCS1,LSS,MVD,MVK,SQLE
**Pathway_SMP00107_Zoledronate Pathway**	ACAT2,FDFT1,FDPS,GGPS1,HMGCR,HMGCS1,LSS,MVD,MVK,SQLE
**Pathway_SMP00111_Cerivastatin Pathway**	ACAT2,FDFT1,FDPS,GGPS1,HMGCR,HMGCS1,LSS,MVD,MVK,SQLE
**Pathway_SMP00112_Risedronate Pathway**	ACAT2,FDFT1,FDPS,GGPS1,HMGCR,HMGCS1,LSS,MVD,MVK,SQLE
**Pathway_SMP00117_Pamidronate Pathway**	ACAT2,FDFT1,FDPS,GGPS1,HMGCR,HMGCS1,LSS,MVD,MVK,SQLE
**Pathway_SMP00119_Fluvastatin Pathway**	ACAT2,FDFT1,FDPS,GGPS1,HMGCR,HMGCS1,LSS,MVD,MVK,SQLE
**Pathway_SMP00131_Atorvastatin Pathway**	ACAT2,FDFT1,FDPS,GGPS1,HMGCR,HMGCS1,LSS,MVD,MVK,SQLE

Table S2. EnrichmentAnalysis_toppCluster_DE_gene downregulated by the L1 24-hour treatment in Caco2 cells

Verbose ID	Caco2_L1_vs_Caco2_non-treated_DOWN_in_Caco2_L1_GeneSet; -LOG10 = 10
**Pathway_1268714_Asparagine N-linked glycosylation**	ALG12,ALG8,AMDHD2,ANK3,ARF1,ARFGAP1,ARFGAP2,ASGR2,B4GALT3,B4GALT5,BET1L,CALR,CANX,CMAS,COG4,COG7,COPA,COPB2,COPG2,CTSA,CTSC,DCTN5,DHDDS,DPAGT1,DPM2,DPM3,DYNC1H1,DYNC1LI2,DYNLL2,ENGASE,FUCA1,FUOM,GANAB,GFUS,GLB1,GMDS,GMPPB,GNPNAT1,KDELR2,LMAN1,MAN1A1,MAN1A2,MAN2A2,MANEA,MGAT3,MGAT5,MIA3,MLEC,MOGS,MPDU1,NANP,NUS1,PREB,PRKCSH,RFT1,RPN1,RPN2,SCFD1,SEC23IP,SEC24C,SERPINA1,SPTBN2,ST3GAL3,ST6GAL1,TRAPPC2L,TRAPPC4,UGGT1,VCP
**Pathway_1268838_Organelle biogenesis and maintenance**	ALMS1,ATAT1,ATP5F1B,B9D1,BBS1,CARM1,CCP110,CCT2,CCT3,CCT4,CCT5,CCT8,CDK1,CEP192,CEP250,CEP41,CEP43,CEP78,CEP83,CKAP5,CYCS,DYNC1H1,DYNC2I2,DYNLL2,EXOC4,EXOC5,EXOC7,GFM1,GFM2,HAUS1,HAUS2,HAUS5,HAUS7,HDAC6,HSP90AA1,HSPB11,IFT122,IFT140,IFT80,IFT81,MAPK12,MAPK14,MRPL1,MRPL17,MRPL19,MRPL20,MRPL21,MRPL3,MRPL30,MRPL36,MRPL37,MRPL38,MRPL39,MRPL4,MRPL46,MRPL47,MRPL52,MRPS15,MRPS16,MRPS17,MRPS18A,MRPS27,MRPS30,MRPS35,MRPS9,NEDD1,PLK4,POLG2,PPARGC1B,PPRC1,PRKAB2,PRKACA,PRKAG1,RPGRIP1L,RXRA,TCP1,TFAM,TFB1M,TTC30A,TUBG1,TUFM,UNC119B,WDR35,YWHAE
**Pathway_1269123_NEP/NS2 Interacts with the Cellular Export Machinery**	NDC1,NUP107,NUP153,NUP155,NUP160,NUP188,NUP205,NUP210,NUP43,NUP58,NUP62,NUP85,NUP98,RANBP2,SEH1L,XPO1
**Pathway_1269654_Transcriptional Regulation by TP53**	AGO1,AKT1,AKT2,BARD1,BAX,CARM1,CCNE1,CCNH,CCNT1,CDK1,CHD4,CHEK2,CNOT1,CNOT9,COX20,COX7B,COX8A,CYCS,E2F1,E2F4,E2F7,EHMT2,ELOA,ERCC2,EXO1,FANCD2,GPI,GSR,GTF2H3,HDAC1,HDAC2,LAMTOR3,LRPPRC,MAPK14,MLST8,MRE11,MSH2,MT-CO1,MT-CO2,MT-CO3,NBN,NDRG1,NDUFA4,NOC2L,NPM1,PCNA,PERP,PIP4K2B,PMS2,POLR2B,POLR2E,POLR2H,PPP2R1B,PRDX1,PRDX2,PRELID1,PRKAA1,PRKAB2,PRKAG1,PRMT1,PRMT5,RABGGTA,RAD51D,RAD9A,RBBP4,RBL2,RFC2,RFC3,RFC4,RFC5,RMI1,RMI2,RPA1,RPTOR,STEAP3,SUPT16H,TAF5,TAF9B,TFDP1,TMEM219,TOP3A,TOPBP1,TSC2,TXN,TXNRD1,USP7,YWHAE
**Pathway_1269688_Processing of Capped Intron-Containing Pre-mRNA**	ALYREF,CASC3,CDC40,CPSF1,CPSF2,CSTF1,CSTF2,CSTF3,CWC27,DDX42,DHX9,DNAJC8,EIF4A3,GCFC2,HNRNPA2B1,HNRNPA3,HNRNPK,HNRNPL,HSPA8,MAGOHB,METTL3,MTREX,NCBP1,NDC1,NUP107,NUP153,NUP155,NUP160,NUP188,NUP205,NUP210,NUP43,NUP58,NUP62,NUP85,NUP98,NXT1,POLR2B,POLR2E,POLR2H,PRPF19,PRPF31,PRPF38A,PTBP1,RANBP2,RNPS1,SEH1L,SF3A3,SF3B1,SF3B3,SF3B6,SLBP,SNRNP200,SNRNP25,SNRPA1,SNU13,SRRM2,SRRT,SRSF1,SRSF10,SRSF2,SRSF3,SRSF6,SYMPK,THOC5,TXNL4A,U2SURP,USP39
**Pathway_1269693_Transport of Mature Transcript to Cytoplasm**	ALYREF,CASC3,CDC40,CPSF1,CPSF2,EIF4A3,MAGOHB,NCBP1,NDC1,NUP107,NUP153,NUP155,NUP160,NUP188,NUP205,NUP210,NUP43,NUP58,NUP62,NUP85,NUP98,NXT1,RANBP2,RNPS1,SEH1L,SLBP,SRSF1,SRSF2,SRSF3,SRSF6,SYMPK,THOC5
**Pathway_1269694_Transport of Mature mRNA derived from an Intron-Containing Transcript**	ALYREF,CASC3,CDC40,EIF4A3,MAGOHB,NCBP1,NDC1,NUP107,NUP153,NUP155,NUP160,NUP188,NUP205,NUP210,NUP43,NUP58,NUP62,NUP85,NUP98,NXT1,RANBP2,RNPS1,SEH1L,SRSF1,SRSF2,SRSF3,SRSF6,THOC5

**Pathway_1269695_Transport of Mature mRNAs Derived from Intronless Transcripts**	ALYREF,CPSF1,CPSF2,NCBP1,NDC1,NUP107,NUP153,NUP155,NUP160,NUP188,NUP205,NUP210,NUP43,NUP58,NUP62,NUP85,NUP98,RANBP2,SEH1L,SLBP,SYMPK
**Pathway_1269696_Transport of the SLBP Dependant Mature mRNA**	ALYREF,NCBP1,NDC1,NUP107,NUP153,NUP155,NUP160,NUP188,NUP205,NUP210,NUP43,NUP58,NUP62,NUP85,NUP98,RANBP2,SEH1L,SLBP
**Pathway_1269697_Transport of the SLBP independent Mature mRNA**	ALYREF,NCBP1,NDC1,NUP107,NUP153,NUP155,NUP160,NUP188,NUP205,NUP210,NUP43,NUP58,NUP62,NUP85,NUP98,RANBP2,SEH1L
**Pathway_1269698_Transport of Mature mRNA Derived from an Intronless Transcript**	ALYREF,CPSF1,CPSF2,NCBP1,NDC1,NUP107,NUP153,NUP155,NUP160,NUP188,NUP205,NUP210,NUP43,NUP58,NUP62,NUP85,NUP98,RANBP2,SEH1L,SYMPK
**Pathway_1269741_Cell Cycle**	AKT1,AKT2,ALMS1,ANAPC15,ANAPC7,BARD1,BRCC3,BUB3,CCNE1,CCNH,CCP110,CDC16,CDC23,CDC25A,CDC45,CDC6,CDK1,CDK4,CDK6,CENPE,CENPK,CENPP,CENPT,CENPU,CENPX,CEP192,CEP250,CEP41,CEP43,CEP78,CHEK2,CKAP5,CLSPN,DHFR,DIDO1,DSN1,DYNC1H1,E2F1,E2F2,E2F4,ERCC6L,ESCO2,EXO1,FBXO5,FEN1,FKBPL,FZR1,GINS1,GMNN,H2AJ,H2AX,H2AZ1,HAUS1,HAUS2,HAUS5,HAUS7,HDAC1,HERC2,HSP90AA1,HSP90AB1,KIF23,LIG1,LPIN3,MAPK1,MCM10,MCM2,MCM3,MCM4,MCM5,MCM6,MCM7,MCM8,MRE11,MSH5,MYC,NBN,NCAPD3,NCAPG,NCAPG2,NCAPH2,NDC1,NDC80,NEDD1,NEK6,NPM1,NUDC,NUP107,NUP153,NUP155,NUP160,NUP188,NUP205,NUP210,NUP43,NUP58,NUP62,NUP85,NUP98,ORC5,ORC6,PCNA,PHF8,PLK4,POLA1,POLD1,POLD3,POLE,PPP1CB,PPP2R1B,PRIM1,PRKACA,PRKCA,PSMA2,PSMA3,PSMA4,PSMB10,PSMB6,PSMC3IP,PSMD1,PSMD11,PSMD12,PSMD14,PSMD2,PSMD5,PSMD6,PSME2,PSME3,PSME4,RAB2A,RAD21,RAD51,RAD9A,RANBP2,RB1,RBBP4,RBL2,RFC2,RFC3,RFC4,RFC5,RMI1,RMI2,RPA1,RRM2,RUVBL2,SEH1L,SKP1,SKP2,SMARCA5,SMC1A,SMC2,SMC3,SMC4,SPC24,SPC25,SUN1,SYCP2,TERF1,TERF2,TERT,TFDP1,TMPO,TOP2A,TOP3A,TOPBP1,TUBG1,TUBGCP3,TUBGCP5,TUBGCP6,TYMS,UBE2C,UBE2N,XPO1,YWHAE,ZW10,ZWINT
**Pathway_1269742_Cell Cycle Checkpoints**	ANAPC15,ANAPC7,BARD1,BRCC3,BUB3,CCNE1,CDC16,CDC23,CDC25A,CDC45,CDC6,CDK1,CHEK2,CLSPN,EXO1,H2AX,HERC2,MCM10,MCM2,MCM3,MCM4,MCM5,MCM6,MCM7,MCM8,MRE11,NBN,ORC5,ORC6,PSMA2,PSMA3,PSMA4,PSMB10,PSMB6,PSMD1,PSMD11,PSMD12,PSMD14,PSMD2,PSMD5,PSMD6,PSME2,PSME3,PSME4,RAD9A,RFC2,RFC3,RFC4,RFC5,RMI1,RMI2,RPA1,TOP3A,TOPBP1,UBE2C,UBE2N,YWHAE
**Pathway_1269753_G2/M Checkpoints**	BARD1,BRCC3,CDC25A,CDC45,CDC6,CDK1,CHEK2,CLSPN,EXO1,H2AX,HERC2,MCM10,MCM2,MCM3,MCM4,MCM5,MCM6,MCM7,MCM8,MRE11,NBN,ORC5,ORC6,PSMA2,PSMA3,PSMA4,PSMB10,PSMB6,PSMD1,PSMD11,PSMD12,PSMD14,PSMD2,PSMD5,PSMD6,PSME2,PSME3,PSME4,RAD9A,RFC2,RFC3,RFC4,RFC5,RMI1,RMI2,RPA1,TOP3A,TOPBP1,UBE2N,YWHAE
**Pathway_1269757_Activation of ATR in response to replication stress**	CDC25A,CDC45,CDC6,CLSPN,MCM10,MCM2,MCM3,MCM4,MCM5,MCM6,MCM7,MCM8,ORC5,ORC6,RAD9A,RFC2,RFC3,RFC4,RFC5,RPA1
**Pathway_1269763_Cell Cycle, Mitotic**	AKT1,AKT2,ALMS1,ANAPC15,ANAPC7,BUB3,CCNE1,CCNH,CCP110,CDC16,CDC23,CDC25A,CDC45,CDC6,CDK1,CDK4,CDK6,CENPE,CENPK,CENPP,CENPT,CENPU,CEP192,CEP250,CEP41,CEP43,CEP78,CKAP5,DHFR,DSN1,DYNC1H1,E2F1,E2F2,E2F4,

	ERCC6L,ESCO2,FBXO5,FEN1,FKBPL,FZR1,GINS1,GMNN,H2AJ,H2AX,H2AZ1,HAUS1,HAUS2,HAUS5,HAUS7,HDAC1,HSP90AA1,HSP90AB1,KIF23,LIG1,LPIN3,MAPK1,MCM10,MCM2,MCM3,MCM4,MCM5,MCM6,MCM7,MCM8,MYC,NCAPD3,NCAPG,NCAPG2,NCAPH2,NDC1,NDC80,NEDD1,NEK6,NUDC,NUP107,NUP153,NUP155,NUP160,NUP188,NUP205,NUP210,NUP43,NUP58,NUP62,NUP85,NUP98,ORC5,ORC6,PCNA,PHF8,PLK4,POLA1,POLD1,POLD3,POLE,PPP1CB,PPP2R1B,PRIM1,PRKACA,PRKCA,PSMA2,PSMA3,PSMA4,PSMB10,PSMB6,PSMD1,PSMD11,PSMD12,PSMD14,PSMD2,PSMD5,PSMD6,PSME2,PSME3,PSME4,RAB2A,RAD21,RANBP2,RB1,RBBP4,RBL2,RFC2,RFC3,RFC4,RFC5,RPA1,RRM2,SEH1L,SKP1,SKP2,SMC1A,SMC2,SMC3,SMC4,SPC24,SPC25,TFDP1,TMPO,TOP2A,TUBG1,TUBGCP3,TUBGCP5,TUBGCP6,TYMS,UBE2C,XPO1,YWHAE,ZW10,ZWINT
**Pathway_1269764_Mitotic G1-G1/S phases**	AKT1,AKT2,CCNE1,CCNH,CDC25A,CDC45,CDC6,CDK1,CDK4,CDK6,DHFR,E2F1,E2F2,E2F4,FBXO5,HDAC1,MCM10,MCM2,MCM3,MCM4,MCM5,MCM6,MCM7,MCM8,MYC,ORC5,ORC6,PCNA,POLA1,POLE,PPP2R1B,PRIM1,PSMA2,PSMA3,PSMA4,PSMB10,PSMB6,PSMD1,PSMD11,PSMD12,PSMD14,PSMD2,PSMD5,PSMD6,PSME2,PSME3,RB1,RBBP4,RBL2,RPA1,RRM2,SKP1,SKP2,TFDP1,TOP2A,TYMS
**Pathway_1269768_G1/S Transition**	AKT1,AKT2,CCNE1,CCNH,CDC25A,CDC45,CDC6,CDK1,CDK4,DHFR,E2F1,FBXO5,MCM10,MCM2,MCM3,MCM4,MCM5,MCM6,MCM7,MCM8,MYC,ORC5,ORC6,PCNA,POLA1,POLE,PPP2R1B,PRIM1,PSMA2,PSMA3,PSMA4,PSMB10,PSMB6,PSMD1,PSMD11,PSMD12,PSMD14,PSMD2,PSMD5,PSMD6,PSME2,PSME3,RB1,RPA1,RRM2,SKP1,SKP2,TFDP1,TYMS
**Pathway_1269772_G1/S-Specific Transcription**	CCNE1,CDC25A,CDC45,CDC6,CDK1,DHFR,E2F1,FBXO5,PCNA,POLA1,RRM2,TFDP1,TYMS
**Pathway_1269773_Activation of the pre-replicative complex**	CDC45,CDC6,MCM10,MCM2,MCM3,MCM4,MCM5,MCM6,MCM7,MCM8,ORC5,ORC6,POLA1,POLE,PRIM1,RPA1
**Pathway_1269774_E2F mediated regulation of DNA replication**	CCNE1,CDC25A,CDC45,CDC6,CDK1,DHFR,E2F1,FBXO5,MCM8,ORC5,ORC6,PCNA,POLA1,PPP2R1B,PRIM1,RB1,RRM2,TFDP1,TYMS
**Pathway_1269777_S Phase**	CCNE1,CCNH,CDC25A,CDC45,CDC6,CDK4,ESCO2,FEN1,FZR1,GINS1,LIG1,MCM2,MCM3,MCM4,MCM5,MCM6,MCM7,MCM8,MYC,ORC5,ORC6,PCNA,POLA1,POLD1,POLD3,POLE,PRIM1,PSMA2,PSMA3,PSMA4,PSMB10,PSMB6,PSMD1,PSMD11,PSMD12,PSMD14,PSMD2,PSMD5,PSMD6,PSME2,PSME3,PSME4,RAD21,RB1,RFC2,RFC3,RFC4,RFC5,RPA1,SKP1,SKP2,SMC1A,SMC3
**Pathway_1269779_Synthesis of DNA**	CDC45,CDC6,FEN1,FZR1,GINS1,LIG1,MCM2,MCM3,MCM4,MCM5,MCM6,MCM7,MCM8,ORC5,ORC6,PCNA,POLA1,POLD1,POLD3,POLE,PRIM1,PSMA2,PSMA3,PSMA4,PSMB10,PSMB6,PSMD1,PSMD11,PSMD12,PSMD14,PSMD2,PSMD5,PSMD6,PSME2,PSME3,PSME4,RB1,RFC2,RFC3,RFC4,RFC5,RPA1
**Pathway_1269781_Switching of origins to a post-replicative state**	CDC6,FZR1,MCM2,MCM3,MCM4,MCM5,MCM6,MCM7,MCM8,ORC5,ORC6,PSMA2,PSMA3,PSMA4,PSMB10,PSMB6,PSMD1,PSMD11,PSMD12,PSMD14,PSMD2,PSMD5,PSMD6,PSME2,PSME3,PSME4,RB1
**Pathway_1269782_Orc1 removal from chromatin**	CDC6,FZR1,MCM2,MCM3,MCM4,MCM5,MCM6,MCM7,MCM8,ORC5,ORC6,PSMA2,PSMA3,PSMA4,PSMB10,PSMB6,PSMD1,PSMD11,PSMD12,PSMD14,PSMD2,PSMD5,PSMD6,PSME2,PSME3,PSME4,RB1
**Pathway_1269784_DNA strand elongation**	CDC45,FEN1,GINS1,LIG1,MCM2,MCM3,MCM4,MCM5,MCM6,MCM7,MCM8,PCNA,POLA1,POLD1,POLD3,PRIM1,RFC2,RFC3,RFC4,RFC5,RPA1
**Pathway_1269794_Regulation of DNA replication**	CDC6,E2F1,E2F2,FZR1,GMNN,MCM10,MCM2,MCM3,MCM4,MCM5,MCM6,MCM7,MCM8,ORC5,ORC6,PSMA2,PSMA3,PSMA4,PSMB10,PSMB6,PSMD1,PSMD11,PSMD12,PSMD14,PSMD2,PSMD5,PSMD6,PSME2,PSME3,PSME4,RB1

**Pathway_1269796_Removal of licensing factors from origins**	CDC6,FZR1,GMNN,MCM10,MCM2,MCM3,MCM4,MCM5,MCM6,MCM7,MCM8,ORC5,ORC6,PSMA2,PSMA3,PSMA4,PSMB10,PSMB6,PSMD1,PSMD11,PSMD12,PSMD14,PSMD2,PSMD5,PSMD6,PSME2,PSME3,PSME4,RB1
**Pathway_1269810_M Phase**	ANAPC15,ANAPC7,BUB3,CDC16,CDC23,CDK1,CENPE,CENPK,CENPP,CENPT,CENPU,CKAP5,DSN1,ERCC6L,FBXO5,H2AJ,H2AX,H2AZ1,KIF23,LPIN3,MAPK1,NCAPD3,NCAPG,NCAPG2,NCAPH2,NDC1,NDC80,NEK6,NUDC,NUP107,NUP153,NUP155,NUP160,NUP188,NUP205,NUP210,NUP43,NUP58,NUP62,NUP85,NUP98,PHF8,PPP2R1B,PRKCA,PSMA2,PSMA3,PSMA4,PSMB10,PSMB6,PSMD1,PSMD11,PSMD12,PSMD14,PSMD2,PSMD5,PSMD6,PSME2,PSME3,PSME4,RAB2A,RAD21,RANBP2,RB1,SEH1L,SMC1A,SMC2,SMC3,SMC4,SPC24,SPC25,TMPO,UBE2C,XPO1,ZW10,ZWINT
**Pathway_1269817_Nuclear Pore Complex (NPC) Disassembly**	CDK1,NDC1,NEK6,NUP107,NUP153,NUP155,NUP160,NUP188,NUP205,NUP210,NUP43,NUP58,NUP62,NUP85,NUP98,RANBP2,SEH1L
**Pathway_1269823_Mitotic Metaphase and Anaphase**	ANAPC15,ANAPC7,BUB3,CDC16,CDC23,CENPE,CENPK,CENPP,CENPT,CENPU,CKAP5,DSN1,ERCC6L,FBXO5,NDC80,NUDC,NUP107,NUP160,NUP43,NUP85,NUP98,PPP2R1B,PSMA2,PSMA3,PSMA4,PSMB10,PSMB6,PSMD1,PSMD11,PSMD12,PSMD14,PSMD2,PSMD5,PSMD6,PSME2,PSME3,PSME4,RAD21,RANBP2,SEH1L,SMC1A,SMC3,SPC24,SPC25,TMPO,UBE2C,XPO1,ZW10,ZWINT
**Pathway_1269825_Mitotic Anaphase**	ANAPC15,ANAPC7,BUB3,CDC16,CDC23,CENPE,CENPK,CENPP,CENPT,CENPU,CKAP5,DSN1,ERCC6L,NDC80,NUDC,NUP107,NUP160,NUP43,NUP85,NUP98,PPP2R1B,PSMA2,PSMA3,PSMA4,PSMB10,PSMB6,PSMD1,PSMD11,PSMD12,PSMD14,PSMD2,PSMD5,PSMD6,PSME2,PSME3,PSME4,RAD21,RANBP2,SEH1L,SMC1A,SMC3,SPC24,SPC25,TMPO,UBE2C,XPO1,ZW10,ZWINT
**Pathway_1269826_Separation of Sister Chromatids**	ANAPC15,ANAPC7,BUB3,CDC16,CDC23,CENPE,CENPK,CENPP,CENPT,CENPU,CKAP5,DSN1,ERCC6L,NDC80,NUDC,NUP107,NUP160,NUP43,NUP85,NUP98,PPP2R1B,PSMA2,PSMA3,PSMA4,PSMB10,PSMB6,PSMD1,PSMD11,PSMD12,PSMD14,PSMD2,PSMD5,PSMD6,PSME2,PSME3,PSME4,RAD21,RANBP2,SEH1L,SMC1A,SMC3,SPC24,SPC25,UBE2C,XPO1,ZW10,ZWINT
**Pathway_1269831_M/G1 Transition**	CDC45,CDC6,E2F1,E2F2,GMNN,MCM10,MCM2,MCM3,MCM4,MCM5,MCM6,MCM7,MCM8,ORC5,ORC6,POLA1,POLE,PRIM1,PSMA2,PSMA3,PSMA4,PSMB10,PSMB6,PSMD1,PSMD11,PSMD12,PSMD14,PSMD2,PSMD5,PSMD6,PSME2,PSME3,PSME4,RPA1
**Pathway_1269832_DNA Replication Pre-Initiation**	CDC45,CDC6,E2F1,E2F2,GMNN,MCM10,MCM2,MCM3,MCM4,MCM5,MCM6,MCM7,MCM8,ORC5,ORC6,POLA1,POLE,PRIM1,PSMA2,PSMA3,PSMA4,PSMB10,PSMB6,PSMD1,PSMD11,PSMD12,PSMD14,PSMD2,PSMD5,PSMD6,PSME2,PSME3,PSME4,RPA1
**Pathway_1269833_Assembly of the pre-replicative complex**	CDC6,E2F1,E2F2,GMNN,MCM2,MCM3,MCM4,MCM5,MCM6,MCM7,MCM8,ORC5,ORC6,PSMA2,PSMA3,PSMA4,PSMB10,PSMB6,PSMD1,PSMD11,PSMD12,PSMD14,PSMD2,PSMD5,PSMD6,PSME2,PSME3,PSME4
**Pathway_1269875_DNA Replication**	CDC45,CDC6,E2F1,E2F2,FEN1,FZR1,GINS1,GMNN,LIG1,MCM10,MCM2,MCM3,MCM4,MCM5,MCM6,MCM7,MCM8,ORC5,ORC6,PCNA,POLA1,POLD1,POLD3,POLE,PRIM1,PSMA2,PSMA3,PSMA4,PSMB10,PSMB6,PSMD1,PSMD11,PSMD12,PSMD14,PSMD2,PSMD5,PSMD6,PSME2,PSME3,PSME4,RB1,RFC2,RFC3,RFC4,RFC5,RPA1
**Pathway_1269957_Metabolism of carbohydrates**	AGL,AGRN,ALDH1A1,B3GAT3,B3GNT3,B4GALT3,B4GALT5,B4GAT1,CALM2,CALM3,CHST12,DCXR,DERA,ENO1,EPM2A,EXT1,GAA,GALE,GALT,GAPDH,GLB1,GLYCTK,GNS,GOT2,GPC3,GPI,GYG2,HEXA,HSPG2,LCT,MAN2B2,MAN2C1,MDH1,MDH2,NDC1,NUP107,NUP153,NUP155,NUP160,NUP188,NUP205,NUP210,NUP43,NUP58,NUP62,NUP85,NUP98,PAPSS1,PFKL,PFKM,PPP2R1B,PRKACA,PRPS2,PYGL,RANBP2,RPE,RPIA,SDC1,SEH1L,SI,SLC25A10,SLC25A11,SLC25A13,SLC26A1,SLC35B3,SLC37A4,SLC5A2,SLC5A9,SORD,ST3GAL3,TALDO1,TKT,VCAN,XYLB

**Pathway_1270121_The citric acid (TCA) cycle and respiratory electron transport**	ACAD9,ACO2,ATP5F1A,ATP5F1B,ATP5F1C,ATP5F1E,ATP5MC1,ATP5MC3,ATP5PB,ATP5PD,BSG,COX20,COX7B,COX8A,CS,CYC1,CYCS,D2HGDH,DLAT,DLD,DMAC2L,ETFA,FH,IDH3A,IDH3B,IDH3G,LDHB,LRPPRC,MDH2,MPC1,MT-ATP6,MT-CO1,MT-CO2,MT-CO3,MT-CYB,MT-ND1,MT-ND2,MT-ND3,MT-ND4,MT-ND5,MT-ND6,NDUFA10,NDUFA11,NDUFA4,NDUFA5,NDUFA7,NDUFA9,NDUFAF3,NDUFAF4,NDUFB1,NDUFB10,NDUFB7,NDUFS1,NDUFS3,NDUFS6,NDUFS7,NDUFS8,NDUFV1,NDUFV2,NDUFV3,NNT,OGDH,PDHA1,PDHX,PDK1,RXRA,SDHA,SDHB,SUCLA2,SUCLG1,SUCLG2,TMEM126B,TRAP1,UCP2,UQCRC1,UQCRC2,UQCRH,UQCRQ
**Pathway_1270122_Pyruvate metabolism and Citric Acid (TCA) cycle**	ACO2,BSG,CS,D2HGDH,DLAT,DLD,FH,IDH3A,IDH3B,IDH3G,LDHB,MDH2,MPC1,NNT,OGDH,PDHA1,PDHX,PDK1,RXRA,SDHA,SDHB,SUCLA2,SUCLG1,SUCLG2
**Pathway_1270125_Citric acid cycle (TCA cycle)**	ACO2,CS,DLD,FH,IDH3A,IDH3B,IDH3G,MDH2,NNT,OGDH,SDHA,SDHB,SUCLA2,SUCLG1,SUCLG2
**Pathway_1270127_Respiratory electron transport, ATP synthesis by chemiosmotic coupling, and heat production by uncoupling proteins.**	ACAD9,ATP5F1A,ATP5F1B,ATP5F1C,ATP5F1E,ATP5MC1,ATP5MC3,ATP5PB,ATP5PD,COX20,COX7B,COX8A,CYC1,CYCS,DMAC2L,ETFA,LRPPRC,MT-ATP6,MT-CO1,MT-CO2,MT-CO3,MT-CYB,MT-ND1,MT-ND2,MT-ND3,MT-ND4,MT-ND5,MT-ND6,NDUFA10,NDUFA11,NDUFA4,NDUFA5,NDUFA7,NDUFA9,NDUFAF3,NDUFAF4,NDUFB1,NDUFB10,NDUFB7,NDUFS1,NDUFS3,NDUFS6,NDUFS7,NDUFS8,NDUFV1,NDUFV2,NDUFV3,SDHA,SDHB,TMEM126B,TRAP1,UCP2,UQCRC1,UQCRC2,UQCRH,UQCRQ
**Pathway_1270128_Respiratory electron transport**	ACAD9,COX20,COX7B,COX8A,CYC1,CYCS,ETFA,LRPPRC,MT-CO1,MT-CO2,MT-CO3,MT-CYB,MT-ND1,MT-ND2,MT-ND3,MT-ND4,MT-ND5,MT-ND6,NDUFA10,NDUFA11,NDUFA4,NDUFA5,NDUFA7,NDUFA9,NDUFAF3,NDUFAF4,NDUFB1,NDUFB10,NDUFB7,NDUFS1,NDUFS3,NDUFS6,NDUFS7,NDUFS8,NDUFV1,NDUFV2,NDUFV3,SDHA,SDHB,TMEM126B,TRAP1,UQCRC1,UQCRC2,UQCRH,UQCRQ
**Pathway_1270133_Metabolism of nucleotides**	ADK,ADSL,ADSS2,AK2,APRT,ATIC,CAD,CTPS1,CTPS2,DCTPP1,DHODH,DUT,ENTPD6,GART,GDA,GMPS,GSR,IMPDH2,LHPP,NT5C,NT5C2,NUDT5,PAICS,PFAS,PNP,PPAT,RRM1,RRM2,TK1,TXN,TXNRD1,TYMS,UCK1
**Pathway_1270135_Purine ribonucleoside monophosphate biosynthesis**	ADSL,ADSS2,ATIC,GART,GMPS,IMPDH2,LHPP,PAICS,PFAS,PPAT
**Pathway_1270158_Metabolism of amino acids and derivatives**	ACAD8,ACADSB,ACAT1,ADO,AFMID,AHCY,AIMP1,ALDH6A1,ALDH7A1,APIP,ASL,ASRGL1,ASS1,BCAT1,BCKDHB,BCKDK,CKMT1B,CNDP2,CPS1,CSAD,DDC,DHTKD1,DLAT,DLD,ENOPH1,EPRS1,ETHE1,FAH,FTCD,GAMT,GCDH,GCSH,GLUD1,GLUL,GOT2,GRHPR,GSR,HAAO,HIBADH,HIBCH,IARS1,INMT,IVD,IYD,KYAT1,KYAT3,LARS1,MAT1A,MCCC1,MCCC2,MTAP,MTR,MTRR,NAALAD2,NMRAL1,OCA2,ODC1,OGDH,PAPSS1,PDHA1,PDHX,PHYKPL,PSMA2,PSMA3,PSMA4,PSMB10,PSMB6,PSMD1,PSMD11,PSMD12,PSMD14,PSMD2,PSMD5,PSMD6,PSME2,PSME3,PSME4,PSTK,PYCR2,PYCR3,QARS1,QDPR,SERINC5,SHMT1,SLC25A10,SLC25A15,SLC7A5,SRM,SUOX,TDO2,TST,TXNRD1

**Pathway_1270161_Branched-chain amino acid catabolism**	ACAD8,ACADSB,ACAT1,ALDH6A1,BCAT1,BCKDHB,BCKDK,DLD,HIBADH,HIBCH,IVD,MCCC1,MCCC2,SHMT1
**Pathway_1270181_Sulfur amino acid metabolism**	ADO,AHCY,APIP,CNDP2,CSAD,ENOPH1,ETHE1,MAT1A,MTAP,MTR,MTRR,SLC25A10,SUOX,TST
**Pathway_1270350_DNA Repair**	ALKBH2,ASCC3,BARD1,BRCC3,CCNH,CENPX,CHD1L,CHEK2,CLSPN,COPS3,COPS6,COPS8,CUL4A,DCLRE1C,DDB1,EME2,ERCC2,ERCC8,EXO1,FAAP100,FANCA,FANCD2,FANCL,FEN1,FTO,GPS1,GTF2H3,H2AX,HERC2,HMGN1,KPNA2,LIG1,LIG3,MGMT,MRE11,MSH2,MSH6,MUS81,NBN,OGG1,PARP2,PCNA,PMS2,PNKP,POLD1,POLD3,POLE,POLH,POLR2B,POLR2E,POLR2H,PPP5C,PRKDC,PRPF19,RAD51,RAD51AP1,RAD51D,RAD9A,RFC2,RFC3,RFC4,RFC5,RIF1,RMI1,RMI2,RPA1,RTEL1,SLX4,SMARCA5,SMUG1,SPIDR,TDG,TDP1,TIPIN,TOP3A,TOPBP1,UBE2N,UBE2T,UNG,USP1,USP10,USP7,VCP,XRCC1,XRCC2,XRCC3,XRCC5
**Pathway_1270351_Base Excision Repair**	FEN1,LIG1,LIG3,OGG1,PARP2,PCNA,PNKP,POLD1,POLD3,POLE,RFC2,RFC3,RFC4,RFC5,RPA1,SMUG1,TDG,UNG,XRCC1
**Pathway_1270359_Resolution of Abasic Sites (AP sites)**	FEN1,LIG1,LIG3,OGG1,PARP2,PCNA,PNKP,POLD1,POLD3,POLE,RFC2,RFC3,RFC4,RFC5,RPA1,SMUG1,TDG,UNG,XRCC1
**Pathway_1270414_Cellular responses to stress**	AGO1,ANAPC15,ANAPC7,ATG3,ATP7A,BAG1,BAG2,BAG4,CCAR2,CCNE1,CDC16,CDC23,CDK4,CDK6,CHMP3,CYBA,CYCS,DYNLL2,E2F1,E2F2,EED,EHMT2,EZH2,FKBP4,FZR1,GSR,H2AJ,H2AX,H2AZ1,HDAC6,HIGD1A,HMGA2,HSBP1,HSP90AA1,HSP90AB1,HSPA14,HSPA4L,HSPA8,HSPA9,HSPH1,ID1,LAMTOR3,MAP2K3,MAPK1,MAPK14,MAPK9,MLST8,MRE11,NBN,NDC1,NUP107,NUP153,NUP155,NUP160,NUP188,NUP205,NUP210,NUP43,NUP58,NUP62,NUP85,NUP98,PRDX1,PRDX2,PRDX3,PRDX6,PRKAA1,PRKAB2,PRKAG1,PSMA2,PSMA3,PSMA4,PSMB10,PSMB6,PSMD1,PSMD11,PSMD12,PSMD14,PSMD2,PSMD5,PSMD6,PSME2,PSME3,PSME4,PTGES3,RANBP2,RB1,RBBP4,RPA1,RPS6KA3,RPTOR,SEH1L,SOD2,SP1,ST13,TERF1,TERF2,TFDP1,TSC2,TXN,TXNRD1,UBE2C,UBN1,VCP,YWHAE
**Pathway_1270421_Cellular response to heat stress**	BAG1,BAG2,BAG4,CCAR2,FKBP4,HDAC6,HSBP1,HSP90AA1,HSP90AB1,HSPA14,HSPA4L,HSPA8,HSPA9,HSPH1,MAPK1,MLST8,NDC1,NUP107,NUP153,NUP155,NUP160,NUP188,NUP205,NUP210,NUP43,NUP58,NUP62,NUP85,NUP98,PTGES3,RANBP2,RPA1,RPTOR,SEH1L,ST13,VCP,YWHAE
**Pathway_1270425_Regulation of HSF1-mediated heat shock response**	BAG1,BAG2,BAG4,CCAR2,HSPA14,HSPA4L,HSPA8,HSPA9,HSPH1,MAPK1,NDC1,NUP107,NUP153,NUP155,NUP160,NUP188,NUP205,NUP210,NUP43,NUP58,NUP62,NUP85,NUP98,RANBP2,RPA1,SEH1L,ST13,YWHAE
**Pathway_1309088_tRNA processing**	ADAT2,CPSF1,CSTF2,DDX1,EPRS1,FAM98B,FTSJ1,METTL1,NDC1,NSUN2,NUP107,NUP153,NUP155,NUP160,NUP188,NUP205,NUP210,NUP43,NUP58,NUP62,NUP85,NUP98,OSGEP,POP1,POP5,POP7,PRORP,QTRT1,QTRT2,RANBP2,RPP14,RPP30,SEH1L,TRIT1,TRMT10C,TRMT6,TRMT61A,TRMU,TRNT1,TSEN54,URM1,WDR4
**Pathway_1309089_tRNA processing in the nucleus**	CPSF1,CSTF2,DDX1,FAM98B,NDC1,NUP107,NUP153,NUP155,NUP160,NUP188,NUP205,NUP210,NUP43,NUP58,NUP62,NUP85,NUP98,POP1,POP5,POP7,RANBP2,RPP14,RPP30,SEH1L,TRNT1,TSEN54
**Pathway_1309095_DNA Double-Strand Break Repair**	BARD1,BRCC3,CHEK2,CLSPN,DCLRE1C,EME2,EXO1,FEN1,H2AX,HERC2,KPNA2,LIG3,MRE11,MUS81,NBN,PARP2,PCNA,POLD1,POLD3,POLE,POLH,PPP5C,PRKDC,RAD51,RAD51AP1,RAD51D,RAD9A,RFC2,RFC3,RFC4,RFC5,RIF1,RMI1,RMI2,RPA1,RTEL1,SLX4,SMARCA5,SPIDR,TDP1,TIPIN,TOP3A,TOPBP1,UBE2N,XRCC1,XRCC2,XRCC3,XRCC5

**Pathway_1309102_HDR through Homologous Recombination (HRR)**	BARD1,EME2,EXO1,MRE11,MUS81,NBN,PCNA,POLD1,POLD3,POLE,POLH,RAD51,RAD51AP1,RAD51D,RAD9A,RFC2,RFC3,RFC4,RFC5,RMI1,RMI2,RPA1,RTEL1,SLX4,SPIDR,TOP3A,TOPBP1,XRCC2,XRCC3
**Pathway_1309103_Homologous DNA Pairing and Strand Exchange**	BARD1,EXO1,MRE11,NBN,RAD51,RAD51AP1,RAD51D,RAD9A,RFC2,RFC3,RFC4,RFC5,RMI1,RMI2,RPA1,TOP3A,TOPBP1,XRCC2,XRCC3
**Pathway_1309105_Resolution of D-Loop Structures**	BARD1,EME2,EXO1,MRE11,MUS81,NBN,RAD51,RAD51AP1,RAD51D,RMI1,RMI2,RTEL1,SLX4,SPIDR,TOP3A,XRCC2,XRCC3
**Pathway_1309117_Transcription-Coupled Nucleotide Excision Repair (TC-NER)**	CCNH,COPS3,COPS6,COPS8,CUL4A,DDB1,ERCC2,ERCC8,GPS1,GTF2H3,HMGN1,LIG1,LIG3,PCNA,POLD1,POLD3,POLE,POLR2B,POLR2E,POLR2H,PRPF19,RFC2,RFC3,RFC4,RFC5,RPA1,USP7,XRCC1
**Pathway_1309120_Gap-filling DNA repair synthesis and ligation in TC-NER**	CCNH,CUL4A,DDB1,ERCC2,ERCC8,GTF2H3,HMGN1,LIG1,LIG3,PCNA,POLD1,POLD3,POLE,POLR2B,POLR2E,POLR2H,PRPF19,RFC2,RFC3,RFC4,RFC5,RPA1,USP7,XRCC1
**Pathway_1339146_Complex I biogenesis**	ACAD9,MT-ND1,MT-ND2,MT-ND3,MT-ND4,MT-ND5,MT-ND6,NDUFA10,NDUFA11,NDUFA5,NDUFA7,NDUFA9,NDUFAF3,NDUFAF4,NDUFB1,NDUFB10,NDUFB7,NDUFS1,NDUFS3,NDUFS6,NDUFS7,NDUFS8,NDUFV1,NDUFV2,NDUFV3,TMEM126B
**Pathway_137934_E2F transcription factor network**	CBX5,CCNE1,CDC25A,CDC6,CDK1,CES2,DHFR,E2F1,E2F2,E2F4,E2F7,HDAC1,MCM3,MYC,POLA1,PRMT5,RANBP1,RB1,RBBP4,RBL2,RRM1,RRM2,SP1,TFDP1,TK1,TOPBP1,TRRAP,TYMS,XRCC1
**Pathway_169351_Validated targets of C-MYC transcriptional activation**	BAX,BCAT1,CAD,CDC25A,CDCA7,CDK4,DDX18,EIF4A1,EIF4G1,ENO1,GAPDH,GPAM,HSP90AA1,HSPD1,HUWE1,MTA1,MTDH,MYC,NBN,NCL,NPM1,ODC1,PEG10,PFKM,PRDX3,PTMA,RIOX2,RUVBL2,SHMT1,TERT,TFRC,TK1,TRRAP
**Pathway_413348_Citrate cycle, second carbon oxidation, 2-oxoglutarate => oxaloacetate**	DLD,FH,MDH1,MDH2,OGDH,OGDHL,SDHA,SDHB,SUCLA2,SUCLG1,SUCLG2
**Pathway_782397_TCA cycle**	ACO2,CS,FH,IDH3A,IDH3G,MDH1,MDH2,OGDH,SDHA,SDHB,SUCLA2,SUCLG1,SUCLG2
**Pathway_790012_Biosynthesis of amino acids**	ACO1,ACO2,ACY1,ALDH7A1,ASL,ASS1,BCAT1,CPS1,CS,ENO1,GAPDH,GLUL,GOT2,IDH3A,IDH3B,IDH3G,MAT1A,MAT2A,MTR,PFKL,PFKM,PRPS2,PYCR2,PYCR3,RPE,RPIA,SHMT1,SHMT2,TALDO1,TKT
**Pathway_814926_Carbon metabolism**	ACADM,ACAT1,ACO1,ACO2,ALDH6A1,CPS1,CS,DLAT,DLD,ECHS1,ENO1,ESD,FH,GAPDH,GLUD1,GLYCTK,GOT2,GPI,HADHA,HIBCH,IDH3A,IDH3B,IDH3G,MDH1,MDH2,ME1,ME2,OGDH,OGDHL,PDHA1,PFKL,PFKM,PRPS2,RPE,RPIA,SDHA,SDHB,SHMT1,SHMT2,SUCLA2,SUCLG1,SUCLG2,TALDO1,TKT

**Pathway_82927_Citrate cycle (TCA cycle)**	ACO1,ACO2,CS,DLAT,DLD,FH,IDH3A,IDH3B,IDH3G,MDH1,MDH2,OGDH,OGDHL,PDHA1,SDHA,SDHB,SUCLA2,SUCLG1,SUCLG2
**Pathway_82942_Oxidative phosphorylation**	ATP5F1A,ATP5F1B,ATP5F1C,ATP5F1E,ATP5MC1,ATP5MC3,ATP5PB,ATP5PD,ATP6V0B,ATP6V1A,ATP6V1D,COX7B,COX8A,CYC1,LHPP,MT-ATP6,MT-CO1,MT-CO2,MT-CO3,MT-CYB,MT-ND1,MT-ND2,MT-ND3,MT-ND4,MT-ND4L,MT-ND5,MT-ND6,NDUFA10,NDUFA11,NDUFA4,NDUFA5,NDUFA7,NDUFA9,NDUFB1,NDUFB10,NDUFB7,NDUFS1,NDUFS3,NDUFS6,NDUFS7,NDUFS8,NDUFV1,NDUFV2,NDUFV3,PPA1,SDHA,SDHB,UQCRC1,UQCRC2,UQCRH,UQCRQ
**Pathway_82952_Valine, leucine and isoleucine degradation**	ACAA1,ACAD8,ACADM,ACADSB,ACAT1,ALDH1B1,ALDH3A2,ALDH6A1,ALDH7A1,BCAT1,BCKDHB,DLD,ECHS1,HADH,HADHA,HADHB,HIBADH,HIBCH,HMGCS2,IVD,MCCC1,MCCC2
**Pathway_82964_Tryptophan metabolism**	ACAT1,AFMID,ALDH1B1,ALDH3A2,ALDH7A1,DDC,ECHS1,GCDH,HAAO,HADH,HADHA,INMT,KYAT1,KYAT3,MAOB,OGDH,OGDHL,TDO2
**Pathway_83039_DNA replication**	FEN1,LIG1,MCM2,MCM3,MCM4,MCM5,MCM6,MCM7,PCNA,POLA1,POLD1,POLD3,POLE,PRIM1,RFC2,RFC3,RFC4,RFC5,RPA1
**Pathway_83054_Cell cycle**	ANAPC13,ANAPC7,BUB3,CCNE1,CCNH,CDC14B,CDC16,CDC23,CDC25A,CDC45,CDC6,CDK1,CDK4,CDK6,CHEK2,E2F1,E2F2,E2F4,FZR1,HDAC1,HDAC2,MCM2,MCM3,MCM4,MCM5,MCM6,MCM7,MYC,ORC5,ORC6,PCNA,PRKDC,RAD21,RB1,RBL2,SKP1,SKP2,SMC1A,SMC3,TFDP1,TGFB2,YWHAE
**Pathway_835393_superpathway of conversion of glucose to acetyl CoA and entry into the TCA cycle**	ACO2,CS,DLAT,DLD,FH,IDH3A,IDH3G,MDH1,MDH2,OGDH,PDHA1,PFKL,PFKM,SDHA,SDHB,SUCLA2,SUCLG1,SUCLG2
**Pathway_855811_Citrate cycle (TCA cycle, Krebs cycle)**	ACO1,ACO2,CS,DLD,FH,IDH3A,IDH3B,IDH3G,MDH1,MDH2,OGDH,OGDHL,SDHA,SDHB,SUCLA2,SUCLG1,SUCLG2
**Pathway_M11835_Valine, leucine and isoleucine degradation**	ACAA1,ACAD8,ACADM,ACADSB,ACAT1,ALDH1B1,ALDH3A2,ALDH6A1,ALDH7A1,BCAT1,BCKDHB,DLD,ECHS1,HADH,HADHA,HADHB,HIBADH,HIBCH,HMGCS2,IVD,MCCC1,MCCC2
**Pathway_M13486_Huntington's disease**	AP2A1,AP2B1,ATP5F1A,ATP5F1B,ATP5F1C,ATP5F1E,ATP5MC1,ATP5MC3,ATP5PB,ATP5PD,BAX,BDNF,CLTC,COX7B,COX8A,CYC1,CYCS,DNAH1,DNAH3,DNAL1,HDAC1,HDAC2,MT-ATP6,MT-CO1,MT-CO2,MT-CO3,MT-CYB,NDUFA10,NDUFA4,NDUFA5,NDUFA7,NDUFA9,NDUFB1,NDUFB10,NDUFB7,NDUFS1,NDUFS3,NDUFS6,NDUFS7,NDUFS8,NDUFV1,NDUFV2,NDUFV3,PLCB3,POLR2B,POLR2E,POLR2H,PPID,SDHA,SDHB,SIN3A,SOD2,SP1,TFAM,TGM2,UQCRC1,UQCRC2,UQCRH,UQCRQ,VDAC1
**Pathway_M16024_Alzheimer's disease**	ATP5F1A,ATP5F1B,ATP5F1C,ATP5F1E,ATP5MC1,ATP5MC3,ATP5PB,ATP5PD,CALM2,CALM3,COX7B,COX8A,CYC1,CYCS,GAPDH,IDE,ITPR3,MAPK1,MT-ATP6,MT-CO1,MT-CO2,MT-CO3,MT-CYB,NAE1,NCSTN,NDUFA10,NDUFA4,NDUFA5,NDUFA7,NDUFA9,NDUFB1,NDUFB10,NDUFB7,NDUFS1,NDUFS3,NDUFS6,NDUFS7,NDUFS8,NDUFV1,NDUFV2,NDUFV3,PLCB3,PPP3CA,PPP3CB,PSENEN,SDHA,SDHB,UQCRC1,UQCRC2,UQCRH,UQCRQ

**Pathway_M16853_DNA replication**	FEN1,LIG1,MCM2,MCM3,MCM4,MCM5,MCM6,MCM7,PCNA,POLA1,POLD1,POLD3,POLE,PRIM1,RFC2,RFC3,RFC4,RFC5,RPA1
**Pathway_M19540_Oxidative phosphorylation**	ATP5F1A,ATP5F1B,ATP5F1C,ATP5F1E,ATP5MC1,ATP5MC3,ATP5PB,ATP5PD,ATP6V0B,ATP6V1A,ATP6V1D,COX7B,COX8A,CYC1,LHPP,MT-ATP6,MT-CO1,MT-CO2,MT-CO3,MT-CYB,MT-ND1,MT-ND2,MT-ND3,MT-ND4,MT-ND4L,MT-ND5,MT-ND6,NDUFA10,NDUFA11,NDUFA4,NDUFA5,NDUFA7,NDUFA9,NDUFB1,NDUFB10,NDUFB7,NDUFS1,NDUFS3,NDUFS6,NDUFS7,NDUFS8,NDUFV1,NDUFV2,NDUFV3,PPA1,SDHA,SDHB,UQCRC1,UQCRC2,UQCRH,UQCRQ
**Pathway_M39335_DNA Replication**	CDC45,CDC6,GMNN,MCM10,MCM2,MCM3,MCM4,MCM5,MCM6,MCM7,ORC5,ORC6,PCNA,POLA1,POLD1,POLD3,POLE,PRIM1,RFC2,RFC3,RFC4,RFC5,RPA1
**Pathway_M39417_Electron Transport Chain (OXPHOS system in mitochondria)**	ATP5F1A,ATP5F1B,ATP5F1C,ATP5F1E,ATP5MC1,ATP5MC3,ATP5PB,ATP5PD,COX7B,COX8A,DMAC2L,MT-ATP6,MT-CO1,MT-CO2,MT-CO3,MT-CYB,MT-ND1,MT-ND2,MT-ND3,MT-ND4,MT-ND4L,MT-ND5,MT-ND6,NDUFA10,NDUFA4,NDUFA5,NDUFA7,NDUFA9,NDUFB1,NDUFB10,NDUFB7,NDUFS1,NDUFS3,NDUFS6,NDUFS7,NDUFS8,NDUFV1,NDUFV2,NDUFV3,SDHA,SDHB,UCP2,UQCRC1,UQCRC2,UQCRH,UQCRQ
**Pathway_M39436_Oxidative phosphorylation**	ATP5F1A,ATP5F1B,ATP5F1E,ATP5MC1,ATP5MC3,ATP5PB,ATP5PD,DMAC2L,MT-ATP6,MT-ND1,MT-ND2,MT-ND3,MT-ND4,MT-ND4L,MT-ND5,MT-ND6,NDUFA10,NDUFA11,NDUFA4,NDUFA5,NDUFA7,NDUFA9,NDUFB1,NDUFB10,NDUFB7,NDUFS1,NDUFS3,NDUFS6,NDUFS7,NDUFS8,NDUFV1,NDUFV2,NDUFV3
**Pathway_M39460_TCA Cycle (aka Krebs or citric acid cycle)**	ACO2,CS,DLD,FH,IDH3A,IDH3B,IDH3G,MDH2,OGDH,SDHA,SDHB,SUCLA2,SUCLG1,SUCLG2
**Pathway_M39471_One Carbon Metabolism**	AHCY,AHCYL2,ATIC,CHDH,DHFR,DNMT1,DNMT3A,FTCD,GART,MAT1A,MTHFD1,MTR,MTRR,SHMT1,SHMT2,TYMS
**Pathway_M39489_TCA Cycle and Deficiency of Pyruvate Dehydrogenase complex (PDHc)**	ACO1,CS,DLAT,DLD,FH,IDH3A,MDH1,OGDH,PDHA1,SDHA,SUCLG2
**Pathway_M39490_DNA IR-damage and cellular response via ATR**	BARD1,BRCC3,CDC45,CDK1,CHEK2,CLK2,CLSPN,E2F1,EXO1,FANCA,FANCD2,FEN1,H2AX,HERC2,MCM2,MRE11,MSH2,NBN,PCNA,PRKDC,RAD51,RAD9A,RECQL,RFWD3,RMI1,RPA1,SMARCC2,SMC1A,SP1,TDP1,TOP3A,TOPBP1,USP1,XRCC5
**Pathway_M39570_Amino Acid metabolism**	ACAA1,ACADM,ACO2,ADH4,ALDH1A1,ALDH7A1,ASS1,BCAT1,CAD,CPS1,CS,DDC,DLD,EPRS1,FAH,FARSB,FH,FTCD,GLUD1,GLUL,GOT2,GSR,HADH,HIBADH,HIBCH,HMGCS2,IARS1,LARS2,MARS2,MCCC1,MDH1,MDH2,ODC1,OGDH,PDHA1,PDHX,SDHA,SRM,SUCLG1,TDO2,VARS1
**Pathway_M39627_G1 to S cell cycle control**	ATF6B,CCNE1,CCNH,CDC25A,CDC45,CDK1,CDK4,CDK6,E2F1,E2F2,MCM2,MCM3,MCM4,MCM5,MCM6,MCM7,MYC,ORC5,ORC6,PCNA,POLE,PRIM1,RB1,RPA1,TFDP1
**Pathway_M39650_Cell Cycle**	ANAPC13,ANAPC7,BUB3,CCNE1,CCNH,CDC14B,CDC16,CDC23,CDC25A,CDC45,CDC6,CDK1,CDK4,CDK6,CHEK2,E2F1,E2F2,E2F4,FZR1,HDAC1,HDAC2,MCM2,MCM3,MCM4,MCM5,MCM6,MCM7,MYC,ORC5,ORC6,PCNA,PRKDC,RAD21,RB1,RBL2,SKP1,SKP2,SMC1A,SMC3,TFDP1,TGFB2,YWHAE

**Pathway_M39668_DNA Mismatch Repair**	EXO1,LIG1,MSH2,MSH6,PCNA,PMS2,POLD1,POLD3,POLE,RFC2,RFC3,RFC4,RFC5,RPA1
**Pathway_M39678_Retinoblastoma Gene in Cancer**	BARD1,CCNE1,CDC25A,CDC45,CDK1,CDK4,CDK6,DHFR,DNMT1,E2F1,E2F2,FAF1,H2AZ1,HDAC1,MCM3,MCM4,MCM6,MCM7,MSH6,MYC,NPAT,PCNA,PLK4,POLA1,POLD3,POLE,PRIM1,PRKDC,RB1,RBBP4,RFC3,RFC4,RFC5,RPA1,RRM1,RRM2,SIN3A,SKP2,SMC1A,SMC2,SMC3,TFDP1,TOP2A,TYMS
**Pathway_M39734_Ciliary landscape**	AFG3L2,AIMP1,ANKS3,ARHGDIA,BBS1,CALM2,CNOT1,CNOT9,COPS3,COPS6,COPS8,CTNNB1,CTSA,DCAF11,DYNC1H1,DYNC2I2,DYNLL2,ECHS1,EIF5B,EXOC4,EXOC5,EXOC7,EXOSC2,EXOSC9,GLB1,HDAC1,HDAC2,HSPB11,HTRA2,IFT122,IFT140,IFT80,IFT81,IQGAP2,LRPPRC,MCM10,MCM2,MCM3,MCM4,MCM5,MCM6,MCM7,MCM8,MSH2,NDUFA5,NDUFA9,NUDC,PGRMC2,PSMD12,RAB2A,RB1,RNGTT,SMC4,TTC30A,UQCC1,USH1C,ZMYND19,ZYG11B
**Pathway_M39740_Metabolic reprogramming in colon cancer**	ACO2,ENO1,FASN,FH,GAPDH,GART,GLUD1,GOT2,GPI,IDH3A,MDH2,PAICS,PDHA1,PFKL,PPAT,PYCR2,RPIA,SDHB,SHMT2,SUCLG2,TALDO1,TKT
**Pathway_M39781_Mitochondrial complex I assembly model OXPHOS system**	ACAD9,FOXRED1,MT-ND1,MT-ND2,MT-ND4,MT-ND4L,MT-ND5,MT-ND6,NDUFA10,NDUFA5,NDUFA7,NDUFAF3,NDUFAF4,NDUFB1,NDUFB10,NDUFB7,NDUFS1,NDUFS3,NDUFS6,NDUFV1,NDUFV2,NDUFV3,TMEM126B,TMEM186
**Pathway_M3985_Citrate cycle (TCA cycle)**	ACO1,ACO2,CS,DLAT,DLD,FH,IDH3A,IDH3B,IDH3G,MDH1,MDH2,OGDH,OGDHL,PDHA1,SDHA,SDHB,SUCLA2,SUCLG1,SUCLG2
**Pathway_M40_E2F transcription factor network**	CBX5,CCNE1,CDC25A,CDC6,CDK1,CES2,DHFR,E2F1,E2F2,E2F4,E2F7,HDAC1,MCM3,MYC,POLA1,PRMT5,RANBP1,RB1,RBBP4,RBL2,RRM1,RRM2,SP1,TFDP1,TK1,TOPBP1,TRRAP,TYMS,XRCC1
**Pathway_M40049_DNA Repair Pathways Full Network**	APEX2,CCNH,CENPX,CUL4A,DCLRE1C,DDB1,ERCC2,ERCC8,EXO1,FAAP100,FANCA,FANCD2,FANCL,FEN1,GTF2H3,H2AX,LIG1,LIG3,MGMT,MRE11,MSH2,MSH6,NBN,OGG1,PARP2,PCNA,PMS2,PNKP,POLD1,POLD3,POLE,POLH,PRKDC,RAD51,RFC2,RFC3,RFC4,RFC5,RPA1,SMUG1,TDG,TERF2,UNG,USP1,XRCC1,XRCC5
**Pathway_M66_Validated targets of C-MYC transcriptional activation**	BAX,BCAT1,CAD,CDC25A,CDCA7,CDK4,DDX18,EIF4A1,EIF4G1,ENO1,GAPDH,GPAM,HSP90AA1,HSPD1,HUWE1,MTA1,MTDH,MYC,NBN,NCL,NPM1,ODC1,PEG10,PFKM,PRDX3,PTMA,RIOX2,RUVBL2,SHMT1,TERT,TFRC,TK1,TRRAP
**Pathway_M7963_Cell cycle**	ANAPC13,ANAPC7,BUB3,CCNE1,CCNH,CDC14B,CDC16,CDC23,CDC25A,CDC45,CDC6,CDK1,CDK4,CDK6,CHEK2,E2F1,E2F2,E2F4,FZR1,HDAC1,HDAC2,MCM2,MCM3,MCM4,MCM5,MCM6,MCM7,MYC,ORC5,ORC6,PCNA,PRKDC,RAD21,RB1,RBL2,SKP1,SKP2,SMC1A,SMC3,TFDP1,TGFB2,YWHAE
**Pathway_MAP00020_Citrate_cycle_TCA_cycle_MAP00020 Citrate cycle TCA cycle**	ACO1,ACO2,CS,DLD,FH,IDH3A,IDH3G,MDH1,MDH2,OGDH,SDHA,SDHB
**Pathway_MAP00280_Valine_leucine_and_isoleucine_d**	ACAA1,ACADM,ALDH1A1,ALDH1B1,ALDH3A2,ALDH6A1,BCKDHB,ECHS1,HADHA,HADHB,HMGCS2,IVD,MCCC1,MCCC2

**egradation_MAP00280 Valine leucine and isoleucine degradation**	
**Pathway_PW:0000026_citrate cycle**	ACO2,CS,DLD,FH,IDH3A,IDH3B,IDH3G,MDH1,MDH2,OGDH,SDHA,SDHB,SUCLA2,SUCLG1,SUCLG2
**Pathway_PW:0000662_mismatch repair pathway**	EXO1,LIG1,MSH2,MSH5,MSH6,PCNA,PMS2,POLD1,POLD3,RFC2,RFC3,RFC4,RFC5,RPA1
**Pathway_SMP00057_Citric Acid Cycle**	ACO2,CS,DLAT,FH,IDH3A,IDH3B,IDH3G,MDH2,OGDH,PDHA1,SDHA,SDHB,SUCLG1

Figure S5. Luna^{FL} analysis of total cell number, viability, and cell size using Trypan Blue staining in Caco2 cells (A-C) and U118 (D-F).

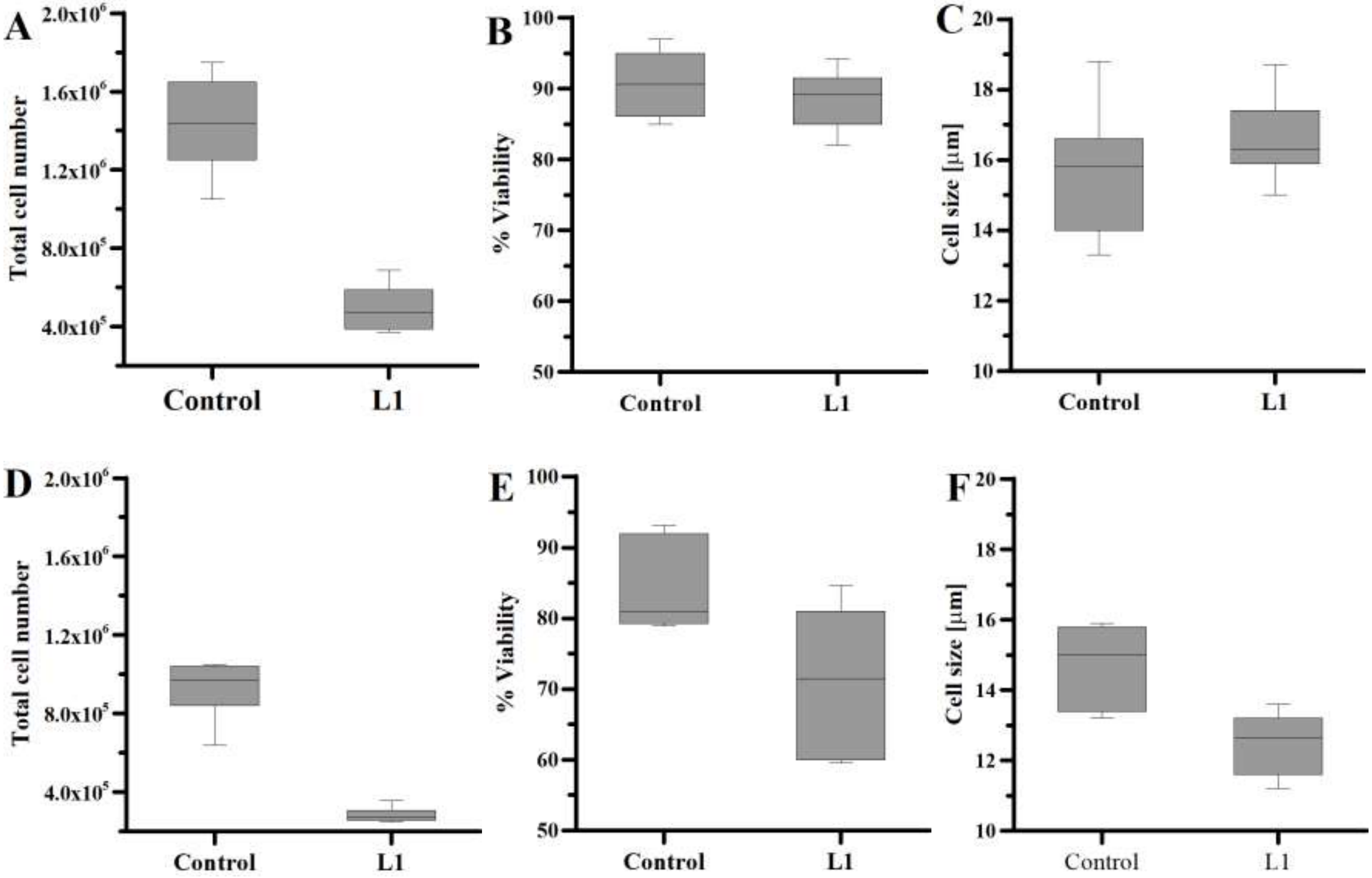

Figure S6. The graphs depict **(A)** the time course of cell number in Caco2 cells treated with L1 (grey colour; L1 concentration: 0.74 mM).) compared to the corresponding control (black colour; untreated cells) as measured by the Luna^{FL} automatic cell counting system. **(B)** The time course of viability percentages in Caco2 cells treated with L1 (grey colour; L1 concentration: 0.74 mM) compared to the corresponding control (black colour; untreated cells) using the Luna^{FL} automatic cell counting system. Data are presented as the mean ± SD (n = 3). $^{\alpha}P < 0.05$, $^{\beta}P < 0.01$; indicating significant differences in cell number compared to the respective values in cells not exposed to L1.

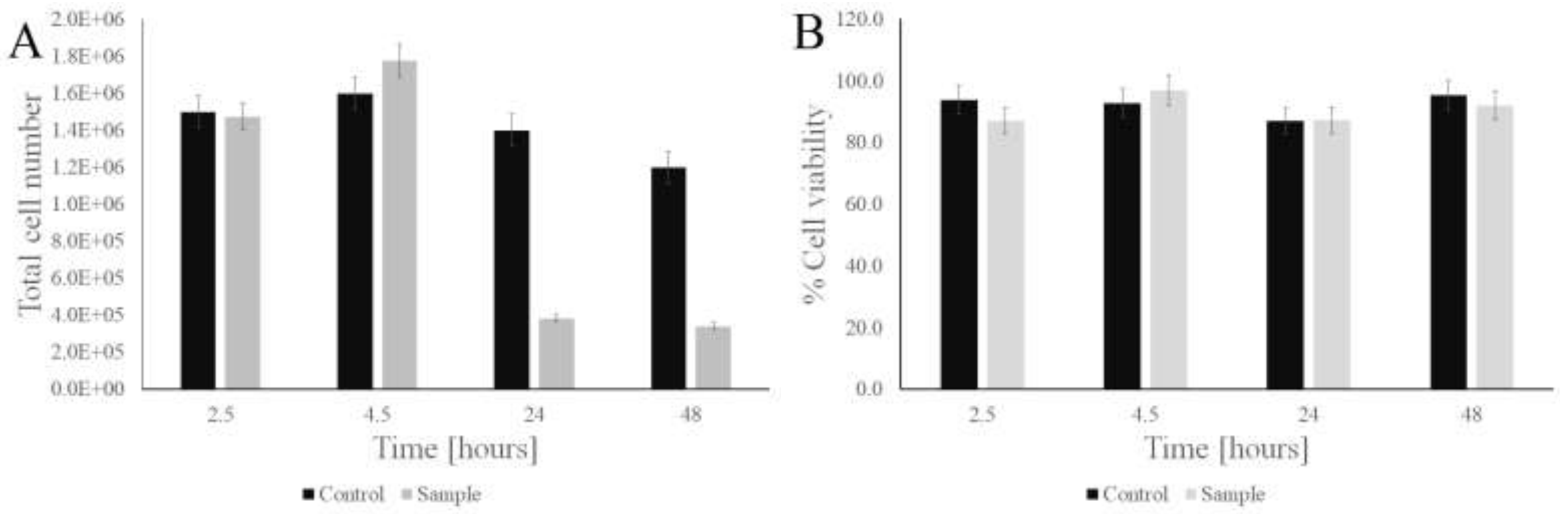

Figure S7. Cytoflow analysis of cell cycle phase distribution (representative data of 3 independent replicates of experiment) in Caco-2 and increasing L1 concentration in cell culture medium (A) 0 mM; (B) 0.7500 mM; (C) 0.3750; (D) 0.1875 mM; (E) 0.0750 mM; (F) 0.0375; (G) 0.0150; (H) 0.0075; (I) 0.0015 mM; (J) 0.0007 mM

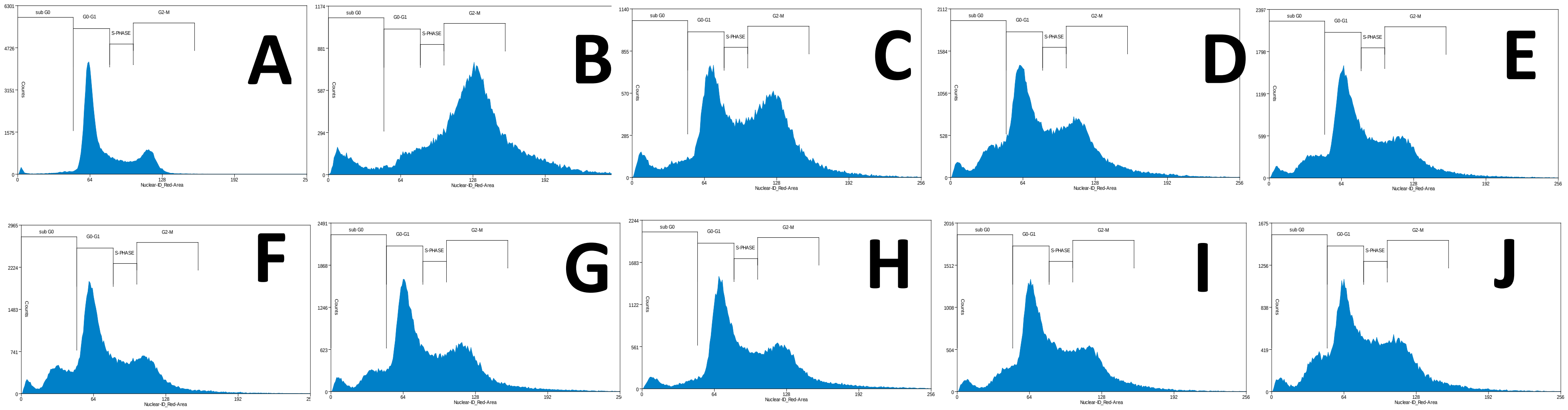

Table S3. Mitotic index calculation.

The mitotic index of control and Colcemid or L1-treated cells was calculated with the use of https://calculator.academy/mitotic-index-calculator/. The total number of cells, as well as number of mitotic cells (G2-M phase) were taken from the Cytoflow analysis with the use of red cell cycle kit (described in section 3.3.3).

**Mitotic index (%)**	**HEK293T**	**Caco2**
Control	10.85	23.36
Nocodazole	55.53	59.68
L1	19.56	32.38
